# Beyond Nutrition: The Therapeutic Promise of Seaweed-Derived Polysaccharides Against Bacterial and Viral Threats

**DOI:** 10.3390/md23100407

**Published:** 2025-10-17

**Authors:** Leonel Pereira, Ana Valado

**Affiliations:** 1 Centre for Functional Ecology (CFE), Marine Resources, Conservation and Technology, Marine Algae Lab, Associate Laboratory TERRA, Department of Life Sciences, University of Coimbra, 3000-456 Coimbra, Portugal; 2 Higher School of Health Technology (ESTESC), Polytechnic University of Coimbra, Rua da Misericórdia, Lagar dos Cortiços, S. Martinho do Bispo, 3045-093 Coimbra, Portugal; 3 Research Center for Natural Resources, Environment and Society (CERNAS), Polytechnic University of Coimbra, Bencanta, 3045-601 Coimbra, Portugal; 4 H&TRC—Health & Technology Research Center, Coimbra Health School, Polytechnic University of Coimbra, Rua 5 de Outubro, 3045-043 Coimbra, Portugal; 5 MARE—Marine and Environmental Sciences Centre/ARNET-Aquatic Research Network, University of Coimbra, 3000-456 Coimbra, Portugal

**Keywords:** seaweed polysaccharides, antibacterial, antiviral, mode of action, carrageenan, agar, fucoidan, alginate, ulvan

## Abstract

In recent years, seaweed-derived polysaccharides have gained recognition as renewed potent bioactive compounds with significant antibacterial and antiviral properties. These polysaccharides include carrageenan, agar, agarose, and porphyran from red seaweed; fucoidan, laminarin, and alginate (alginic acid) from brown seaweed; and ulvan from green seaweed. Their diverse and complex structures, shaped by sulfation patterns, glycosidic linkages, and monosaccharide composition, contribute to their broad-spectrum biological activities, including antimicrobial, immunomodulatory, and prebiotic functions. This review explores the structural characteristics of these marine polysaccharides, reported in vitro and in vivo antimicrobial activities, and the mechanisms underlying their antibacterial and antiviral effects. Additionally, the extraction, purification methods, and commercial applications of these bioactive polysaccharides are discussed. By integrating recent advances and highlighting their multifunctionality, this review underscores the translational promise of seaweed-derived polysaccharides as sustainable, natural agents in the global fight against antimicrobial resistance and infectious diseases.

## 1. Introduction

The global rise in antimicrobial resistance and the emergence of novel viral pathogens have intensified the search for alternative therapeutic agents capable of combating microbial threats. In this context, marine ecosystems have emerged as a rich reservoir of bioactive compounds, with seaweed—also known as marine macroalgae—gaining increasing attention for their pharmacological potential [1]. Traditionally consumed for their nutritional value, macroalgae are now recognized as sources of structurally diverse polysaccharides that exhibit a wide range of biological activities beyond basic nutrition [2].

Seaweed-derived polysaccharides, such as carrageenan, agar, agarose, and porphyran from red algae; fucoidan, laminarin, and alginate from brown algae; and ulvan from green algae possess unique chemical architectures shaped by their monosaccharide composition, degree of sulfation, and glycosidic linkages [3]. These structural features are directly linked to their bioactivity, enabling interactions with microbial membranes, immune cells, and host receptors. As a result, these polysaccharides have demonstrated promising antibacterial and antiviral properties, as well as immunomodulatory and prebiotic effects, both in vitro and in vivo studies [4].

Recent advances in extraction and purification technologies have facilitated the isolation of these compounds with enhanced bioactivity and stability, paving the way for their incorporation into functional foods, pharmaceuticals, and biomedical formulations [5]. Moreover, their biocompatibility, biodegradability, and low toxicity make them attractive candidates for therapeutic applications aimed at infection prevention and immune support [6].

### 1.1. Overview of Seaweed-Derived Polysaccharides and Their Growing Significance

Marine macroalgae, commonly known as seaweed, have long been appreciated for their ecological roles and nutritional value, particularly in coastal communities where they have served as staple foods and agricultural resources [7]. In recent decades, however, seaweed has emerged as a promising source of bioactive compounds with diverse applications in biotechnology, pharmaceuticals, and functional foods. Among these compounds, seaweed-derived polysaccharides stand out due to their structural complexity, biological versatility, and growing relevance in health-related research [8].

These polysaccharides are synthesized as structural and storage molecules within the cell walls of seaweed and exhibit remarkable diversity depending on the algal group. Red algae (Rhodophyta) produce carrageenan, agar, agarose, and porphyran; brown algae (Phaeophyceae) yield fucoidan, laminarin, and alginate; and green algae (Chlorophyta) are known for ulvan [9]. Each of these polysaccharides possesses unique physicochemical properties shaped by their monosaccharide composition, degree and pattern of sulfation, molecular weight, and glycosidic linkages. These structural features are directly linked to their biological activity, influencing their solubility, viscosity, and interaction with biological membranes and receptors [10].

Traditionally, seaweed polysaccharides have been utilized as gelling, stabilizing, and thickening agents in the food, cosmetic, and textile industries [11]. However, recent scientific advances have revealed their potential as therapeutic agents, particularly in the context of antimicrobial, antiviral, immunomodulatory, antioxidant, anticoagulant, and prebiotic functions. This shift from industrial additives to bioactive ingredients is driven by several converging factors. First, the rise in antibiotic resistance and the emergence of novel viral pathogens have created an urgent need for alternative, natural compounds with broad-spectrum antimicrobial activity [12]. Second, the biocompatibility, biodegradability, and low toxicity of seaweed polysaccharides make them attractive candidates for biomedical applications, including drug delivery systems, wound healing materials, and vaccine adjuvants. Third, the sustainable and renewable nature of seaweed cultivation aligns with global efforts to develop environmentally friendly and ethically sourced therapeutic agents [13].

Moreover, the mechanisms by which these polysaccharides exert their biological effects are increasingly being elucidated. Sulfated polysaccharides, for example, have demonstrated the ability to inhibit viral attachment and replication by mimicking host cell surface receptors, thereby blocking viral entry [14]. Others have shown antibacterial activity through disruption of microbial membranes or modulation of host immune responses. In addition, their prebiotic properties contribute to gut health by selectively stimulating beneficial microbiota, which in turn supports immune function and metabolic balance [15].

The growing body of research on seaweed-derived polysaccharides underscores their potential to address pressing health challenges through natural and sustainable means. As interest in marine biopolymers continues to expand, these compounds are poised to play a pivotal role in the development of next-generation therapeutics and functional products [16]. Their integration into multidisciplinary research frameworks—spanning marine biology, pharmacology, immunology, and food science—will be essential for unlocking their full potential and translating laboratory findings into real-world applications [17].

### 1.2. Importance of Investigating Their Antimicrobial and Antiviral Properties

The increasing prevalence of infectious diseases and the global rise in antimicrobial resistance have created an urgent demand for new therapeutic agents that are both effective and sustainable [18]. Conventional antibiotics and antiviral drugs, while historically successful, are now facing serious limitations due to the emergence of resistant microbial strains and novel viral pathogens. This growing challenge has prompted researchers to explore alternative sources of bioactive compounds, particularly those derived from natural ecosystems [19]. Among these, seaweed-derived polysaccharides have emerged as highly promising candidates, owing to their unique structural features and broad-spectrum biological activities [20].

Polysaccharides extracted from marine macroalgae possess a range of physico-chemical properties—such as high molecular weight, sulfation, and complex glycosidic linkages—that enable them to interact with microbial membranes, inhibit viral replication, and modulate host immune responses [21]. Sulfated polysaccharides like carrageenan, fucoidan, and ulvan have demonstrated potent antiviral effects by blocking viral attachment and entry into host cells, mimicking cellular receptors, and interfering with replication cycles. Their antibacterial activity has also been well documented, with mechanisms including membrane disruption, inhibition of biofilm formation, and stimulation of immune defenses [22].

Investigating the antimicrobial and antiviral properties of these compounds is not only scientifically relevant but also strategically important for public health. Seaweed-derived polysaccharides offer several advantages over synthetic drugs: they are biocompatible, biodegradable, and generally exhibit low toxicity. Their natural origin and renewable sourcing from marine environments align with global efforts to develop environmentally friendly and ethically sourced therapeutic agents. These attributes make them attractive for integration into a wide range of applications, including topical formulations, oral supplements, wound dressings, and vaccine adjuvants [8,23].

**Figure 1:**
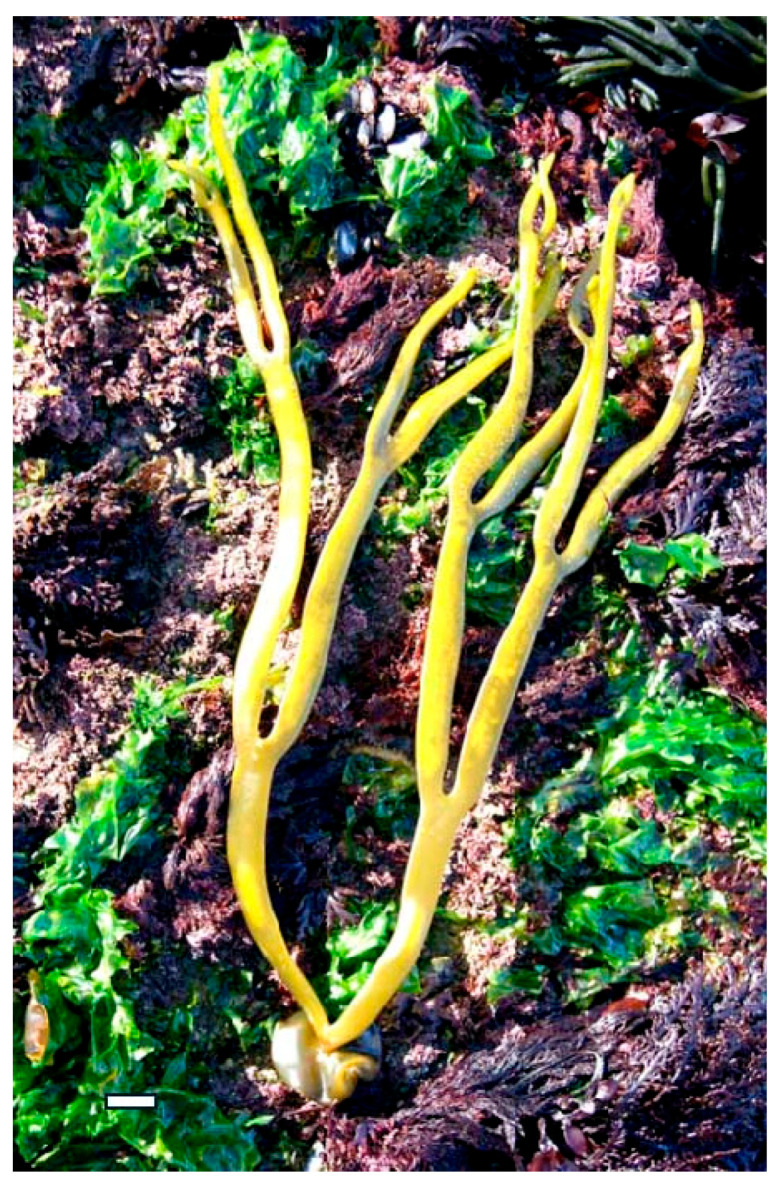
*Himanthalia elongata* (Phaeophyceae), a brown alga rich in bioactive compounds that contribute to oxidative stress modulation, inflammation control, and gut microbiota balance. These properties support healthy aging and may reduce the risk of chronic diseases such as cardiovascular conditions, cancer, and metabolic syndrome (Scale = 1 cm) (image from the authors).

Moreover, exploring these compounds contributes to the diversification of the global pharmacopeia, reducing dependency on conventional antibiotics and expanding the arsenal of tools available to combat infectious diseases. Research continues to uncover the mechanisms underlying their bioactivity, seaweed polysaccharides may play a pivotal role in shaping the future of antimicrobial and antiviral therapy—particularly in the development of multifunctional agents that combine therapeutic efficacy with immunomodulatory and prebiotic benefits. A deeper understanding of their potential not only enhances our knowledge of marine biochemistry but also opens new avenues for translational research, clinical innovation, and the development of nature-based solutions to some of the most pressing health challenges of our time [24,25]. For example, *Himanthalia elongata* (Phaeophyceae) (Figure 1) boasts a rich array of bioactive compounds, granting exceptional versatility in promoting health. Its diverse biochemical profile enables it to modulate oxidative stress, inflammation, and gut microbiota, supporting healthy aging and reducing the risk of chronic conditions such as cardiovascular disease, cancer, and metabolic syndrome.

Given the global rise in antimicrobial resistance and the limitations of conventional therapies, seaweed-derived polysaccharides offer a compelling alternative. Their broad-spectrum antibacterial and antiviral activities, mediated through membrane disruption, viral entry inhibition, and immune modulation, position them as multifunctional agents with low toxicity and high biocompatibility. These compounds are increasingly being explored for integration into wound dressings, oral supplements, and vaccine adjuvants. As natural, renewable resources, they align with sustainable therapeutic development and may play a pivotal role in diversifying the global pharmacopeia. Continued investigation into their mechanisms and applications will be essential for translating marine biochemistry into innovative clinical solutions.

### 1.3. Literature Search Strategy

To ensure a comprehensive and methodologically sound synthesis of the current literature on the antimicrobial and antiviral properties of seaweed-derived polysaccharides, a structured literature search was conducted. This process aimed to identify peer-reviewed articles, reviews, and experimental studies that addressed biological activities, mechanisms of action, and therapeutic applications of polysaccharides extracted from red, brown, and green macroalgae.

The search was performed across several major scientific databases, including PubMed, Scopus, Web of Science, ScienceDirect, and Google Scholar. These platforms were selected to ensure broad coverage of biomedical, marine biotechnology, and interdisciplinary research. Search queries were formulated using combinations of relevant keywords and Boolean operators, such as “seaweed polysaccharides” AND “antibacterial,” “fucoidan” OR “carrageenan” AND “antiviral activity,” “macroalgae” AND “bioactive compounds” AND “infection,” and “ulvan” AND “immune modulation.” This strategy allowed for the retrieval of studies spanning molecular characterization, in vitro and in vivo assays, and translational applications.

Criteria were defined to prioritize articles published between 2000 and 2025 that reported experimental data or mechanistic insights into the antimicrobial or antiviral effects of seaweed-derived polysaccharides. Studies focusing on structural characterization, extraction and purification methods, or therapeutic relevance were considered essential. Only English-language publications were included to maintain consistency in interpretation and synthesis. Conversely, studies lacking experimental validation, mechanistic depth, or biomedical relevance were excluded, as were non-peer-reviewed sources unless cited for historical or contextual purposes.

Titles and abstracts were initially screened for relevance, followed by full-text review of selected articles. Reference lists of key publications were manually examined to identify additional sources that may have been missing during the database search. Emphasis was placed on recent studies and those offering mechanistic clarity or translational perspectives, particularly in the context of infection control, immune modulation, and biomedical applications.

This literature search strategy ensured a balanced and evidence-based foundation for the review, enabling a critical appraisal of the therapeutic promise of seaweed-derived polysaccharides in combating bacterial and viral threats.

## 2. Structural Characteristics of Seaweed Polysaccharides

### 2.1. Classification Based on Seaweed Type (Red, Brown, Green)

Seaweed-derived polysaccharides are structurally diverse macromolecules that play crucial roles in the physiology and ecological adaptation of marine macroalgae. These polysaccharides are synthesized as structural components of the cell wall or as storage molecules, and their chemical complexity is shaped by the species of algae from which they originate [26]. The three major groups of seaweed—red (Rhodophyta), brown (Phaeophyceae), and green (Chlorophyta)—each produce distinct classes of polysaccharides with unique monosaccharide compositions, glycosidic linkages, degrees of sulfation, and molecular weights. These structural features not only determine their physicochemical properties, such as solubility, viscosity, and gel-forming capacity, but also influence their biological activities, including antimicrobial, antiviral, immunomodulatory, and antioxidant effects [27].

**Figure 2:**
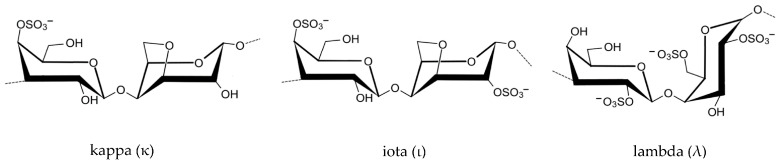
Chemical structures of the main commercial types of carrageenan—kappa (κ), iota (ι), and lambda (λ)—derived from red algae. Each type consists of alternating units of D-galactose and 3,6-anhydro-D-galactose, distinguished by specific sulfation patterns that influence their gelling behavior and biological activity (images from the authors).

Red algae are particularly rich in sulfated galactans, with carrageenan, agar, agarose, and porphyran being the most prominent examples [28]. Carrageenan is composed of alternating units of D-galactose and 3,6-anhydro-D-galactose, with varying sulfation patterns that give rise to different types—kapa (κ), Iota (ι), and lambda (λ) carrageenans (Figure 2)—each with distinct gelling and bioactive properties [29].

**Figure 3:**
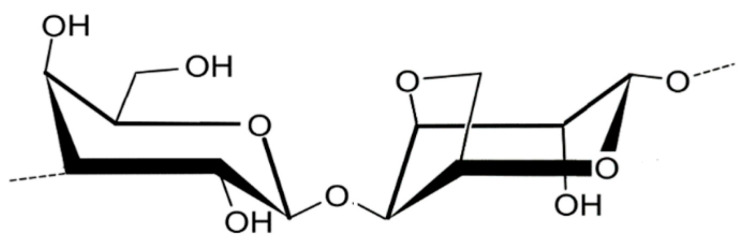
Chemical structure of agar, a polysaccharide composed of repeating agarobiose units—disaccharides of D-galactose and 3,6-anhydro-L-galactose. Agar is widely used in microbiology and biomedical applications due to their strong gel-forming capacity and excellent biocompatibility (image from the authors).

**Figure 4:**
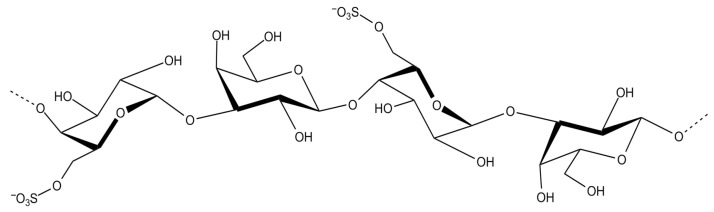
Chemical structure of porphyran, a sulfated galactan found in red algae of the *Porphyra*/*Pyropia* genus. Its unique sulfation pattern contributes to notable antioxidant, anti-inflammatory, and antiviral activities, making it a promising candidate for functional food and nutraceutical applications (image from the authors).

Agar (Figure 3) is built from repeating units of agarobiose, a disaccharide of D-galactose and 3,6-anhydro-L-galactose and are widely used in microbiology and biomedical applications due to their strong gel-forming ability and biocompatibility [30]. Porphyran (Figure 4), found in *Porphyra*/*Pyropia* species, contains sulfated galactose units and has attracted attention for its antioxidant, anti-inflammatory, and antiviral properties, particularly in functional food and nutraceutical contexts [31].

**Figure 5:**
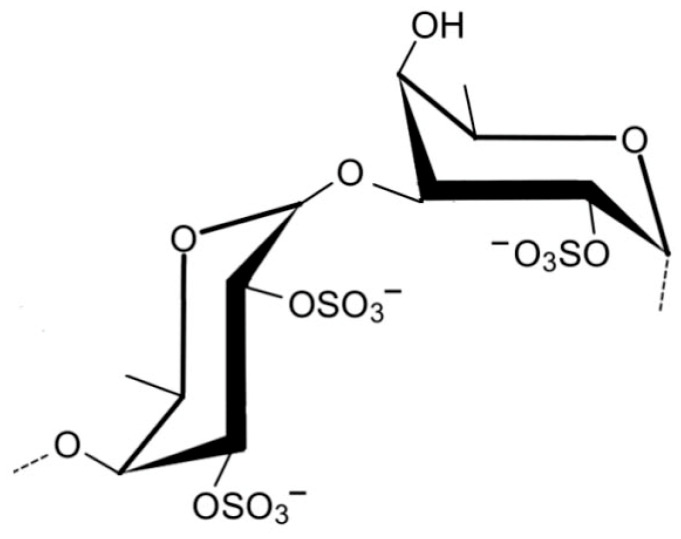
Chemical structure of fucoidan, a highly sulfated polysaccharide predominantly composed of L-fucose, with additional residues of galactose, mannose, xylose, and uronic acids. Its branched and heterogeneous architecture underpins a wide range of biological activities, including antiviral, anticoagulant, and immunomodulatory effects (image from the authors).

Brown algae produce a different set of polysaccharides, including fucoidan, laminarin, and alginate, each with distinct structural and functional characteristics [32]. Fucoidan (Figure 5) is a highly sulfated polysaccharide primarily composed of L-fucose, often accompanied by galactose, mannose, xylose, and uronic acids. Its branched and heterogeneous structure contributes to its potent biological activities, including antiviral, anticoagulant, and immunomodulatory effects [33].

**Figure 6:**
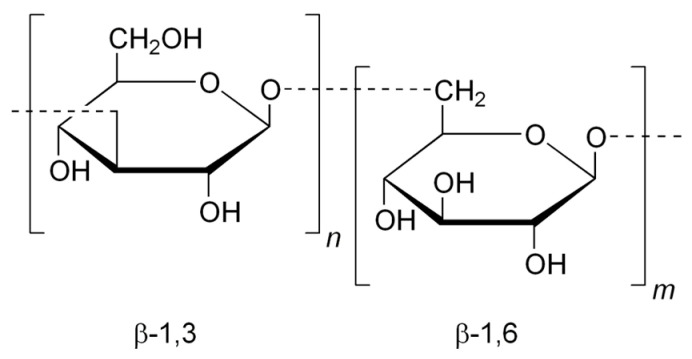
Chemical structure of laminarin, a storage β-glucan composed primarily of glucose units linked by β(1→3) and β(1→6) glycosidic bonds. This polysaccharide has shown immunostimulatory and antimicrobial activities, with promising applications in gut health and wound healing: Edgar 181.

Laminarin (Figure 6) is a storage β-glucan consisting mainly of glucose units linked by β(1→3) and β(1→6) bonds. It has demonstrated immunostimulatory and antimicrobial properties, particularly in gut health and wound healing applications [34].

**Figure 7:**
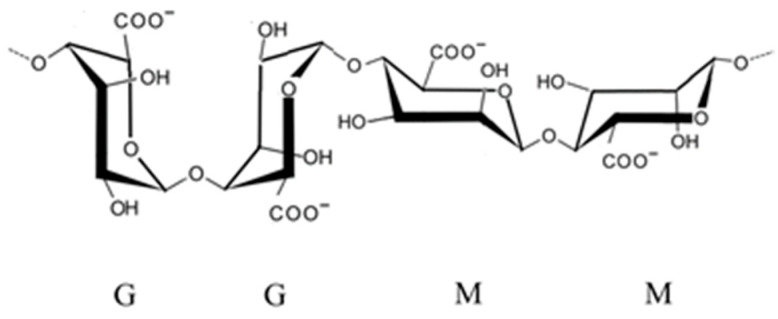
Chemical structure of alginic acid, a linear copolymer composed of β-D-mannuronic acid (M) and α-L-guluronic acid (G) residues. Upon binding with sodium or calcium ions, alginic acid forms alginate salts capable of creating hydrogels—an essential feature for applications in drug delivery, tissue engineering, and wound care due to their biocompatibility and controlled-release properties (image from the authors).

When the alginic acid (Figure 7) binds with sodium and calcium ions, the resulting salts are known as alginate. Alginate is known for its ability to form hydrogels in the presence of divalent cations such as calcium and sodium. This property makes alginate valuable in drug delivery systems, tissue engineering, and wound care products, where controlled release and biocompatibility are essential [35].

**Figure 8:**
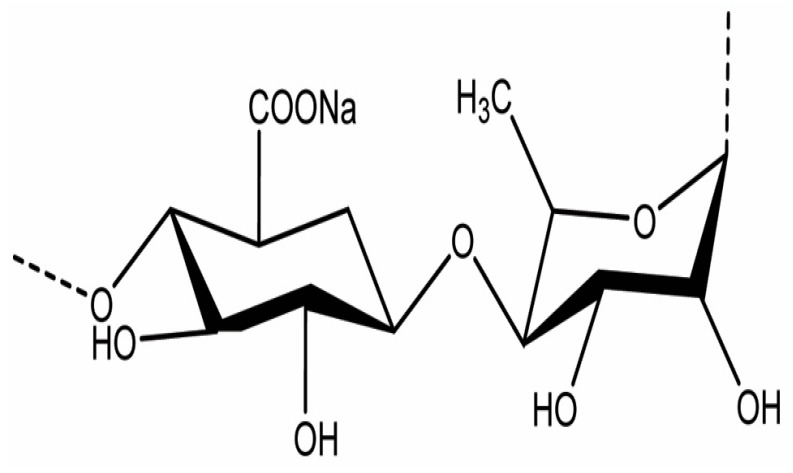
Chemical structure of ulvan, a sulfated heteropolysaccharide primarily extracted from *Ulva* species. Composed of rhamnose, xylose, glucuronic acid, and iduronic acid, ulvan structurally resembles glycosaminoglycans found in animal connective tissues. This similarity underpins its antioxidant, antiviral, and immunomodulatory properties, supporting its potential use in nutraceuticals, pharmaceuticals, and regenerative medicine (adapted from [36]).

Green algae, though less extensively studied, also produce bioactive polysaccharides with promising therapeutic potential. The most notable among them is ulvan, a sulfated heteropolysaccharide extracted primarily from *Ulva* species. Ulvan (Figure 8 is composed of rhamnose, xylose, glucuronic acid, and iduronic acid, and its structure bears resemblance to glycosaminoglycans found in animal connective tissues. This similarity contributes to its antioxidant, antiviral, and immunomodulatory properties, making ulvan a candidate for applications in nutraceuticals, pharmaceuticals, and regenerative medicine. Its ability to form viscous solutions and interact with biological membranes, further enhances its potential as a functional ingredient in health-promoting formulations [36,37].

Structural characteristics of seaweed-derived polysaccharides are deeply influenced by the taxonomy of the source algae. Each class—red, brown, and green—offers a unique set of molecular architectures that underpin their diverse biological functions [38]. Understanding these structural nuances is essential for optimizing extraction and purification methods, enhancing therapeutic efficacy, and tailoring applications in antimicrobial and antiviral strategies. As research continues to unravel the complexity of these marine biopolymers, their role in advancing natural medicine and sustainable health solutions becomes increasingly evident [39].

### 2.2. Chemical Composition and Sulfation Patterns

The chemical composition and sulfation patterns of seaweed-derived polysaccharides are central to their biological activity and therapeutic potential. These macromolecules are composed of various monosaccharides—such as galactose, glucose, mannose, rhamnose, xylose, and uronic acids—linked through glycosidic bonds that vary in position, configuration, and branching [40]. The presence, density, and location of sulfate groups (–SO_3_^−^) on these sugar residues significantly influence the polysaccharide’s solubility, molecular conformation, and ability to interact with microbial surfaces and host receptors. These structural features are not uniform across species but are shaped by the taxonomy of the algae, environmental conditions, and extraction methods [41,42].

In red algae, species such as *Kappaphycus alvarezii*, *Chondrus crispus* and *Mastocarpus stellatus* are widely used for the extraction of carrageenan. This sulfated galactan is typically obtained through hot water extraction followed by alkali treatment to enhance gel strength and remove impurities. The degree of sulfation varies among κ-, ι-, and λ-carrageenan, influencing their antiviral activity [43]. For example, λ-carrageenan, with its higher sulfate content, has demonstrated significant inhibition against enveloped viruses such as *Herpes simplex* virus (HSV) and influenza virus by blocking viral attachment and entry into host cells. The negatively charged sulfate groups mimic host cell surface glycosaminoglycans, preventing viral particles from binding to their natural receptors [43].

**Figure 9:**
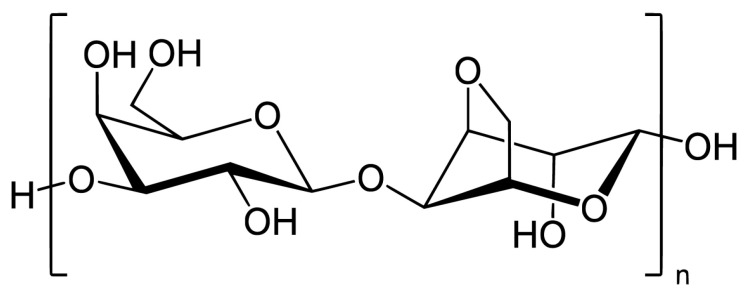
Chemical structure of agarose, a purified fraction of agar extracted from *Gelidium*, *Gelidium amansii*, and *Gracilariopsis longissima* (Rhodophyta). Composed of repeating agarobiose units, agarose exhibits antibacterial activity by disrupting biofilm formation and increasing membrane permeability, particularly in Gram-positive bacteria. (Image in public domain; author: Yikrazuul).

Agarose (Figure 9), extracted from *Gelidium corneum*, *Gelidium amansii* and *Gracilariopsis longissima* (Rhodophyta), are obtained via autoclaving or pressure cooking followed by filtration and freeze–thaw purification. While less sulfated than carrageenan, agarose has shown antibacterial effects by disrupting bacterial biofilm formation and enhancing the permeability of bacterial membranes, particularly in Gram-positive strains [44,45].

Brown algae such as *Fucus vesiculosus* and *Undaria pinnatifida* are rich sources of fucoidan, a highly sulfated fucose-based polysaccharide. Extraction typically involves aqueous or acidic solvents at moderate temperatures, followed by ethanol precipitation and dialysis [46]. The sulfate groups in fucoidan contribute to its broad-spectrum antiviral activity, including inhibition of Human immunodeficiency viruses (HIV), dengue virus, and hepatitis-B virus. Fucoidan interferes with viral replication and modulates immune responses by enhancing macrophage activation and cytokine production. Additionally, its antibacterial properties are linked to its ability to destabilize bacterial membranes and inhibit quorum sensing, thereby reducing virulence and biofilm formation. *F. vesiculosus*, commonly found along temperate coastlines, is one of the most prominent sources of fucoidan due to its high polysaccharide yield, extensive biochemical characterization, and widespread commercial availability, making it a model species for both therapeutic research and industrial extraction [47].

Laminarin, extracted, for example, from *Laminaria digitata* and *Saccharina japonica* (Phaeophyceae), is a β-glucan with minimal sulfation. It is typically isolated using hot water extraction followed by ethanol precipitation [48]. Though less potent as a direct antimicrobial agent, laminarin exhibits immunostimulatory effects by activating Toll-like receptors (TLRs) and promoting phagocytosis, thereby enhancing the host’s innate defense against bacterial infections [49].

**Figure 10:**
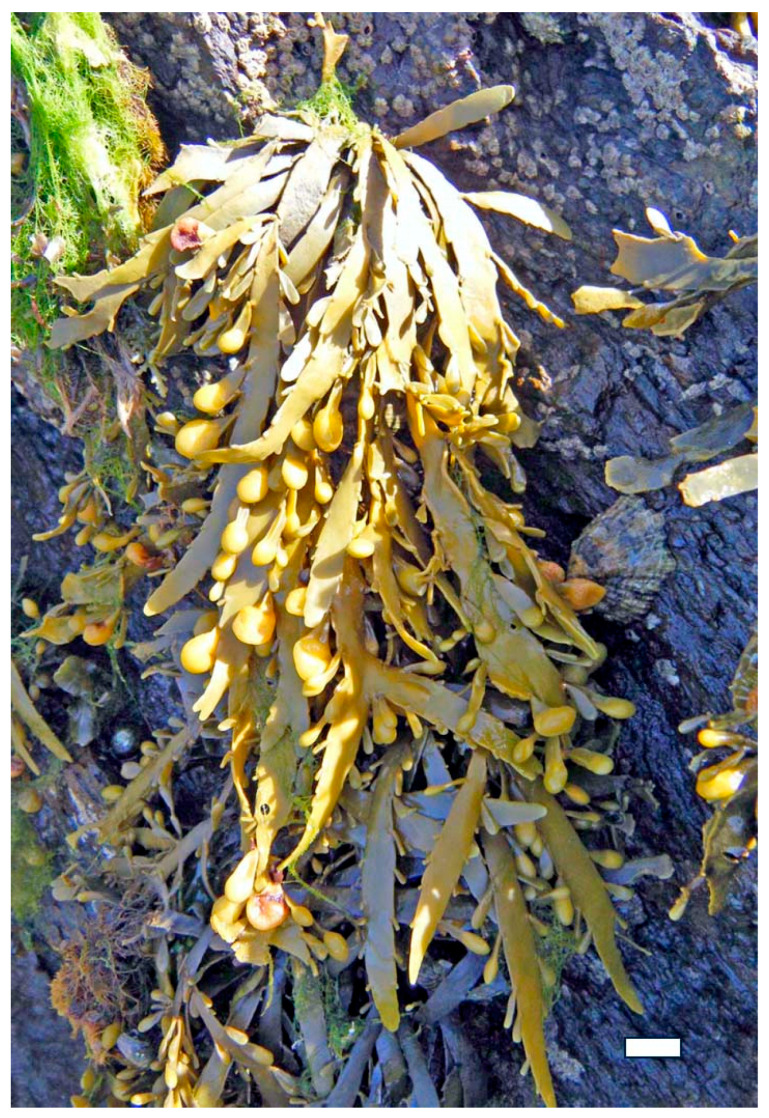
*Ascophyllum nodosum* (Phaeophyceae), one of the primary brown algae sources of alginate. This species is commonly harvested for its high alginic acid content, which is extracted and processed into sodium alginate for biomedical and pharmaceutical applications. (Scale = 1 cm) (Scale = 1 cm) (image from the authors).

Alginate, derived, for example, from *Macrocystis pyrifera* and *Ascophyllum nodosum* (Phaeophyceae) (Figure 10), is a linear copolymer of mannuronic and guluronic acids. Extraction involves alkaline treatment followed by calcium precipitation and acid conversion to sodium alginate [50]. While alginate itself lacks sulfation, its carboxyl groups contribute to its antimicrobial properties by chelating essential metal ions and disrupting microbial metabolism. Alginate-based hydrogels have also been used as carriers for antimicrobial agents, enhancing their delivery and efficacy in wound healing applications [51].

**Figure 11:**
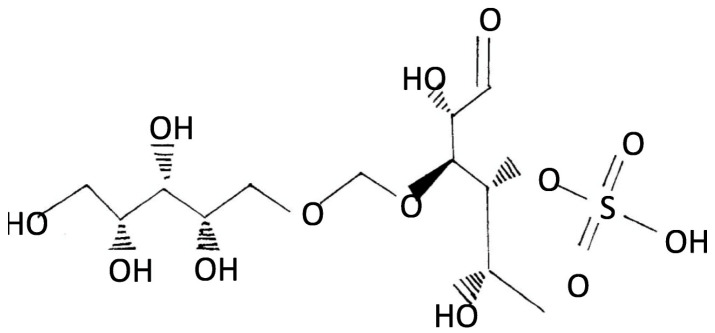
Chemical structure of ascophyllan, a fucose-rich, sulfated polysaccharide uniquely extracted from *Ascophyllum nodosum*. Although structurally related to fucoidans, ascophyllan features a distinct monosaccharide composition and lower molecular weight, contributing to its demonstrated antibacterial activity against *Staphylococcus aureus* and *Escherichia coli* in vitro (image from the authors).

Ascophyllan (Figure 11) is a fucose-rich, sulfated polysaccharide uniquely extracted from *A. nodosum*. While it shares structural similarities with conventional fucoidans, its monosaccharide composition and chemical architecture are notably distinct. This low molecular weight polysaccharide, ascophyllan, possesses in vitro antibacterial activities against the pathogenic bacteria *Staphylococcus aureus* and *Escherichia coli* [52].

**Figure 12:**
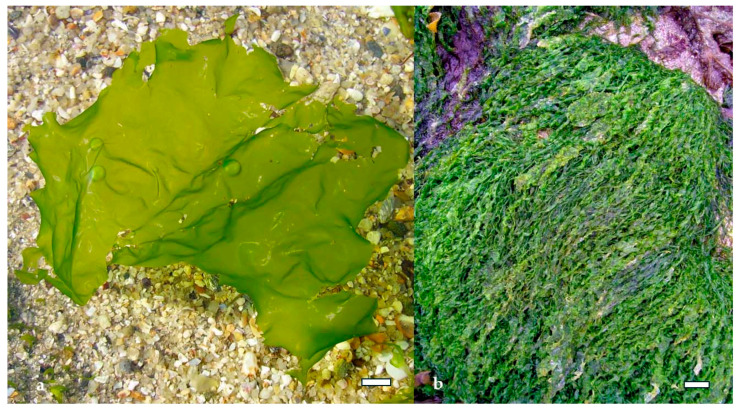
*Ulva lactuca* (**a**) *and Ulva compressa* (**b**) (Chlorophyta), two green algae species known for producing ulvan—a sulfated heteropolysaccharide with structural similarity to glycosaminoglycans. These species are commonly used in ulvan extraction for applications in antiviral and antibacterial therapies (Scale = 1 cm) (images from the authors).

Green algae such as *Ulva lactuca* and *Ulva compressa* (Figure 12) produce ulvan, a sulfated heteropolysaccharide composed of rhamnose, xylose, glucuronic acid, and iduronic acid. Extraction typically involves hot water or dilute acid treatment, followed by ethanol precipitation and ultrafiltration [53]. The sulfation of ulvan occurs mainly on rhamnose residues, and its structural similarity to mammalian glycosaminoglycans enhances its interaction with immune cells. Ulvan has demonstrated antiviral activity against Newcastle disease virus and antibacterial effects against *Staphylococcus aureus* and *Escherichia coli*, primarily through membrane destabilization and immunomodulation [53].

Chemical composition and sulfation patterns of seaweed polysaccharides are intricately linked to their antimicrobial and antiviral mechanisms. Species-specific structural features, combined with optimized extraction techniques, determine the efficacy and applicability of these compounds in therapeutic contexts. Understanding these relationships is essential for harnessing the full potential of marine polysaccharides in combating microbial threats and advancing natural health solutions [3,22].

### 2.3. Structure-Activity Relationships Relevant to Bioactivity

The biological activity of seaweed-derived polysaccharides is deeply rooted in their molecular architecture. Structure–activity relationships (SARs) provide critical insight into how specific chemical features—such as monosaccharide composition, glycosidic linkages, molecular weight, degree of branching, and sulfation patterns—govern the interaction of these macromolecules with microbial pathogens and host cells. These relationships are not only fundamental to understanding their antimicrobial and antiviral mechanisms but also essential for guiding extraction, modification, and formulation strategies aimed at enhancing therapeutic efficacy [54,55].

One of the most influential structural determinants is the degree and position of sulfation, which imparts a negative charge to the polysaccharide and enables electrostatic interactions with positively charged viral proteins and bacterial surfaces. For example, *K. alvarezii*, a red alga widely cultivated for carrageenan production, yields κ-carrageenan with sulfate groups primarily at the C-4 position of galactose residues. This configuration allows κ-carrageenan to inhibit viral entry by mimicking host cell surface glycosaminoglycans, effectively blocking viruses such as HSV and human papillomavirus (HPV). In contrast, λ-carrageenan, with three sulfate groups per disaccharide unit, exhibits even stronger antiviral activity due to its higher charge density and ability to interfere with viral adsorption and replication [56].

**Figure 13:**
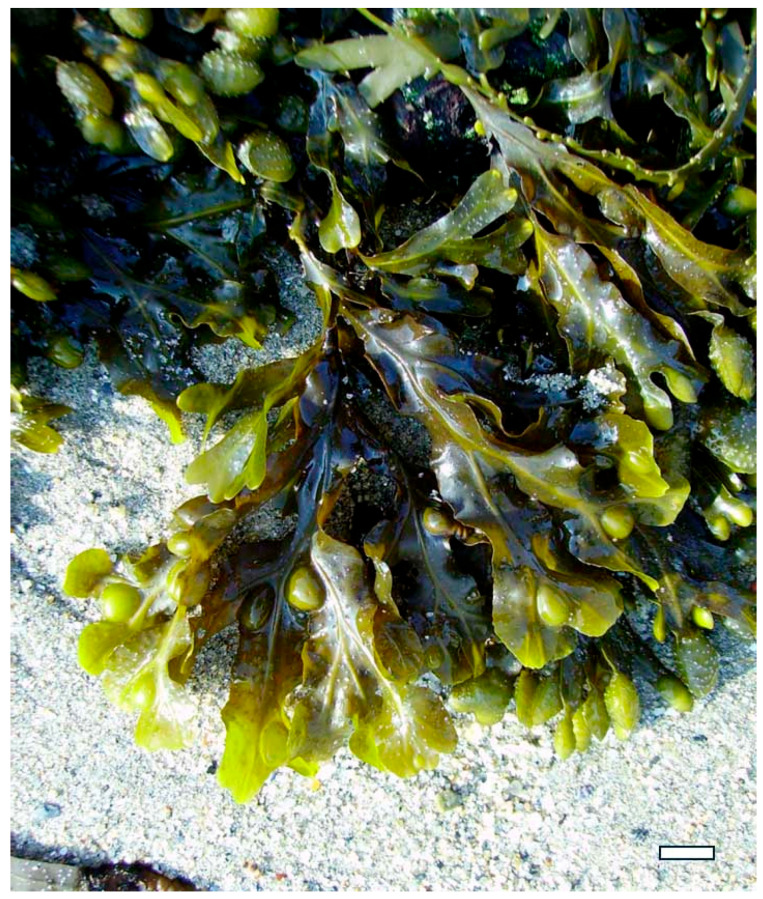
*Fucus vesiculosus* (Phaeophyceae), a brown alga known for its high fucoidan content. The structural complexity of its sulfated polysaccharides contributes to potent antiviral and antibacterial activities, including inhibition of viral replication and disruption of bacterial biofilms (Scale = 1 cm) (image from the authors).

Similarly, *Fucus vesiculosus* (Figure 13), a brown alga rich in fucoidan, produces a highly branched sulfated polysaccharide composed mainly of L-fucose, with sulfate groups variably positioned on C-2 and C-4. This structural heterogeneity enhances fucoidan’s ability to bind viral glycoproteins and inhibit the replication of enveloped viruses such as HIV, influenza, and dengue virus. Fucoidan also exhibits antibacterial activity by disrupting bacterial membrane integrity and inhibiting biofilm formation, particularly in Gram-negative strains like *Escherichia coli* and *Pseudomonas aeruginosa* [57].

Molecular weight is another key factor influencing bioactivity. High-molecular-weight polysaccharides tend to form viscous solutions and gels, which can act as physical barriers to microbial invasion. For example, alginate extracted from *M. pyrifera* forms hydrogels that are effective in wound dressings, where they provide a moist environment and inhibit bacterial colonization. Conversely, low-molecular-weight fractions of fucoidan have demonstrated enhanced cellular uptake and immunostimulatory effects, making them suitable for oral or injectable formulations aimed at systemic immune modulation [58].

**Figure 14:**
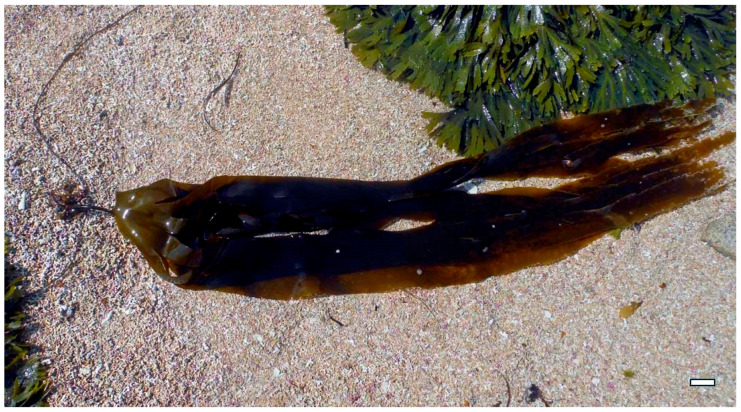
*Laminaria digitata* (Phaeophyceae), a brown alga and primary source of laminarin—a β-glucan composed mainly of glucose units. Laminarin activates innate immune receptors such as Dectin-1 and Toll-like receptors (TLRs), promoting phagocytosis and cytokine production to enhance host defense against bacterial infections (Scale = 1 cm) (image from the authors).

Monosaccharide composition and glycosidic linkages also play a pivotal role in determining biological specificity. Laminarin derived from *Laminaria digitata* (Figure 14), consists mainly of glucose units and activates innate immune receptors such as Dectin-1 and TLRs. This interaction promotes phagocytosis and cytokine production, enhancing the host’s defense against bacterial infections [58].

Ulvan has shown antiviral activity against Newcastle disease virus and antibacterial effects against *Staphylococcus aureus*, primarily through membrane destabilization and immune activation [59].

Branching and conformational flexibility further influence bioactivity. Highly branched polysaccharides offer increased surface area for interaction with microbial membranes and host receptors. Fucoidan’s branched architecture enhances its ability to interfere with bacterial quorum sensing and inhibit virulence factor expression [60]. Meanwhile, the triple-helical conformation of κ-carrageenan contributes to its thermal stability and resistance to enzymatic degradation, making it suitable for sustained-release formulations [61].

Finally, functional groups such as carboxyl and hydroxyl moieties contribute to metal ion chelation, hydrogen bonding, and pH modulation. Alginate’s carboxyl groups allow it to bind divalent cations like calcium and zinc, which are essential for microbial metabolism. This chelation disrupts enzymatic activity and inhibits bacterial growth, particularly in wound environments where alginate-based dressings are commonly applied [62].

## 3. Mechanisms of Antibacterial and Antiviral Action

### 3.1. Cellular Interactions and Inhibition of Pathogen Adhesion

One of the primary mechanisms by which seaweed-derived polysaccharides exert antibacterial and antiviral effects is through direct cellular interactions that interfere with pathogen adhesion to host tissues. Adhesion is a critical first step in the infection process, allowing bacteria and viruses to anchor themselves to epithelial surfaces, initiate colonization, and evade immune clearance. By disrupting this process, polysaccharides act as biological shields, preventing the establishment and progression of infection [63].

**Figure 15:**
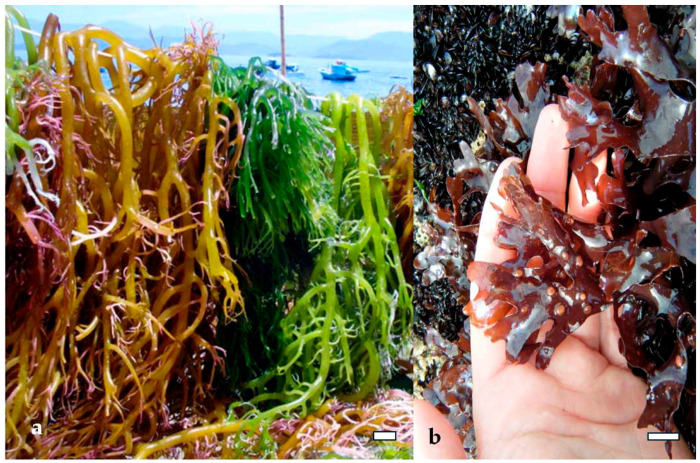
*Kappaphycus alvarezii* (**a**) and *Chondrus crispus* (**b**) (Rhodophyta), two red algae species commonly used for carrageenan extraction. Carrageenan, particularly the λ-type with high sulfate content, exhibits potent antiviral activity by mimicking host glycosaminoglycans and blocking viral attachment to cell receptors (Scale = 1 cm) (images from the authors).

Sulfated polysaccharides, in particular, play a pivotal role in this mechanism due to their high negative charge density, which enables them to mimic host cell surface glycosaminoglycans (GAGs). These GAGs are commonly exploited by pathogens as docking sites for attachment. For example, carrageenan extracted from *Kappaphycus alvarezii* (Figure 15a) and *Chondrus crispus crispus* (Rhodophyta) (Figure 15b) has demonstrated strong antiviral activity against HPV, HSV, and influenza virus by competitively binding to viral envelope glycoproteins [64,65,66]. This prevents the virus from interacting with its natural receptors on host cells, effectively blocking entry and subsequent replication. λ-carrageenan, with its high sulfate content, has shown particular efficacy in inhibiting viral adhesion through electrostatic repulsion and steric hindrance [67,68].

**Figure 16:**
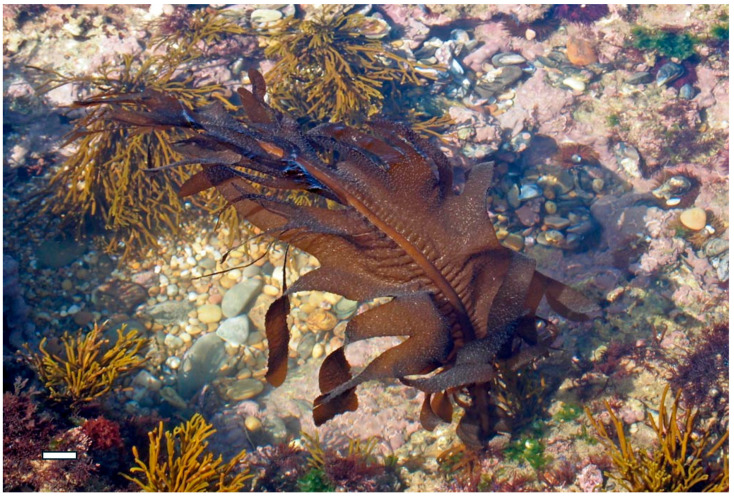
*Undaria pinnatifida* mixed with *Bifurcaria bifurcata* (Phaeophyceae)*,* two brown algae species known for their fucoidan content. Fucoidan extracted from these sources exhibits potent antiviral activity by binding viral particles and modulating host cell receptors, thereby preventing pathogen attachment and enhancing epithelial barrier function (Scale = 1 cm) (image from the authors).

Similarly, fucoidan from *Fucus vesiculosus* and *Undaria pinnatifida* (Figure 16) exhibits potent antiviral effects by binding to viral particles and obstructing their attachment to host cells [69]. Fucoidan’s branched structure and variable sulfation patterns enhance its ability to interact with a wide range of viral glycoproteins, including those of HIV, dengue virus, and hepatitis B virus. In addition to direct viral inhibition, fucoidan has been shown to modulate host cell surface receptors, reducing their availability for pathogen binding and thereby reinforcing the barrier function of epithelial tissues [70].

In the context of bacterial infections, polysaccharides such as ulvan from *U. lactuca* [71] and alginate from *M. pyrifera* [72] interfere with bacterial adhesion by altering surface charge interactions and disrupting biofilm formation. Ulvan’s sulfated rhamnose residues interact with bacterial adhesins, preventing attachment to mucosal surfaces. This mechanism is particularly effective against Gram-positive bacteria like *Staphylococcus aureus*, which rely heavily on surface proteins for colonization [59]. Alginate, although not sulfated, contains carboxyl groups that chelate essential metal ions and disrupt bacterial metabolism, indirectly impairing adhesion and growth [73,74].

Laminarin from *L. digitata*, a β-glucan with minimal sulfation, contributes to antibacterial defense by enhancing host immune recognition. It binds to pattern recognition receptors such as Dectin-1 and TLRs, stimulating the production of antimicrobial peptides and promoting phagocytosis. This immunostimulatory effect strengthens the host’s ability to clear pathogens before they can establish a foothold [75].

### 3.2. Immunomodulatory Effects and Antiviral Pathways

Beyond their direct antimicrobial actions, seaweed-derived polysaccharides exert profound immunomodulatory effects that contribute to their antiviral efficacy. These effects are mediated through interactions with innate and adaptive immune components, enhancing host defense mechanisms and modulating inflammatory responses. The structural complexity of these polysaccharides—particularly their sulfation patterns, molecular weight, and monosaccharide composition—plays a critical role in determining their immunological impact [25].

Sulfated polysaccharides such as fucoidan from *Fucus vesiculosus* and *Undaria pinnatifida* have been extensively studied for their ability to activate immune cells, including macrophages, dendritic cells, and natural killer (NK) cells [76]. Fucoidan binds to pattern recognition receptors (PRRs) such as TLRs and scavenger receptors on immune cells, triggering intracellular signaling cascades that lead to the production of cytokines, chemokines, and interferons. These mediators enhance the antiviral state of host cells, inhibit viral replication, and promote the clearance of infected cells. Fucoidan has also been shown to increase the expression of major histocompatibility complex (MHC) class I and II molecules, thereby improving antigen presentation and stimulating adaptive immune responses [77].

Carrageenan, particularly the λ-type extracted from *C. crispus* and from *Tichocarpus crinitus*, has demonstrated immunomodulatory activity by stimulating the release of type I interferons and pro-inflammatory cytokines such as IL-6 and tumor necrosis factor-alpha (TNF-α) [78]. These cytokines play a central role in orchestrating antiviral defenses, including the activation of cytotoxic T lymphocytes and the inhibition of viral protein synthesis [79]. In addition, carrageenan has been shown to enhance mucosal immunity when applied topically, making it a promising candidate for nasal sprays and vaginal gels aimed at preventing viral transmission [80,81,82].

Ulvan from *U. lactuca* also exhibits immunostimulatory properties, largely attributed to its structural resemblance to glycosaminoglycans found in mammalian tissues. Ulvan interacts with immune receptors on epithelial and immune cells, promoting the secretion of IL-10 and interferon gamma (IFN-γ), which are key regulators of antiviral immunity and inflammation resolution. Its ability to modulate both pro- and anti-inflammatory pathways makes ulvan particularly valuable in managing viral infections that involve immune dysregulation, such as influenza and respiratory syncytial virus (RSV) [83,84].

Laminarin from *L. digitata*, though minimally sulfated, activates innate immune responses through β-glucan receptors such as Dectin-1. This interaction enhances phagocytosis, oxidative burst, and the production of antimicrobial peptides, contributing to the containment and elimination of viral particles [85]. Laminarin also supports the maturation of dendritic cells and the activation of T-helper cells, bridging innate and adaptive immunity [86].

The antiviral pathways influenced by these polysaccharides are multifaceted. They include inhibition of viral entry through receptor blockade, suppression of viral replication via interferon signaling, and enhancement of immune surveillance through cytokine modulation and antigen presentation. Importantly, these effects are not limited to a single virus type but have been observed across a broad spectrum of enveloped and non-enveloped viruses, including herpesviruses, retroviruses, coronaviruses, and orthomyxoviruses [87].

Immunomodulatory effects of seaweed-derived polysaccharides significantly amplify their antiviral potential. By engaging and regulating key components of the immune system, these marine biopolymers offer a dual-action approach—direct viral inhibition and host immune enhancement. Their integration into therapeutic strategies, whether as standalone agents or adjuvants, holds promise for improving outcomes in viral infections and reducing reliance on conventional antivirals [88].

### 3.3. Influence on Gut Microbiota and Prebiotic Benefits

Seaweed-derived polysaccharides are increasingly recognized not only for their antimicrobial and immunomodulatory properties but also for their capacity to modulate gut microbiota and act as natural prebiotics [89]. These complex carbohydrates resist digestion in the upper gastrointestinal tract and reach the colon intact, where they serve as fermentable substrates for beneficial microbes. By selectively stimulating the growth of health-promoting bacterial genera—such as *Bifidobacterium*, *Lactobacillus*, *Faecalibacterium*, and *Akkermansia*—these polysaccharides contribute to microbial balance, enhance short-chain fatty acid (SCFA) production, and support mucosal immunity [90].

**Figure 17:**
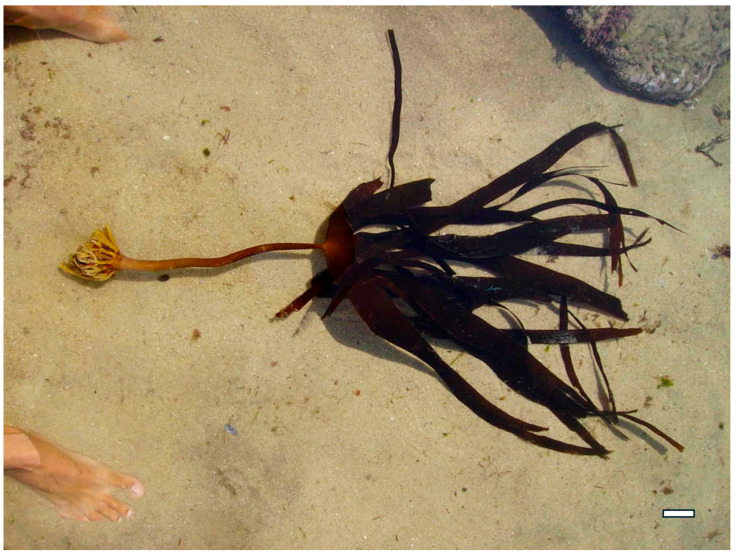
*Laminaria hyperborea* (Phaeophyceae), a brown alga widely used for alginate extraction. Alginate from this species exhibits prebiotic effects by promoting beneficial gut microbiota such as Bacteroides and *Prevotella* and contributes to microbial fermentation and short-chain fatty acid (SCFA) production (Scale = 1 cm) (images from the authors).

Polysaccharides from brown algae are particularly well-studied in this context. For instance, alginate extracted from *A. nodosum* and *Laminaria hyperborea* (Figure 17) have demonstrated prebiotic effects by increasing populations of *Bacteroides* and *Prevotella*, which are involved in carbohydrate fermentation and SCFA synthesis [91]. Alginate’s carboxyl-rich structure allows it to form viscous gels in the gut, slowing nutrient absorption and providing a sustained substrate for microbial fermentation. Additionally, alginate has been shown to bind bile acids and reduce intestinal pH, creating an environment less favorable to pathogenic bacteria [92].

Fucoidan, derived from species such as *Fucus serratus* and *Sargassum fusiforme*, also exhibits promising prebiotic potential. Although its fermentability depends on molecular weight and sulfation degree, fucoidan has been shown to increase the abundance of *Akkermansia muciniphila*, a mucin-degrading bacterium associated with improved metabolic health and reduced inflammation. In animal models, fucoidan supplementation has led to enhanced gut barrier function and reduced endotoxemia, suggesting a role in preventing systemic inflammation linked to dysbiosis [93].

**Figure 18:**
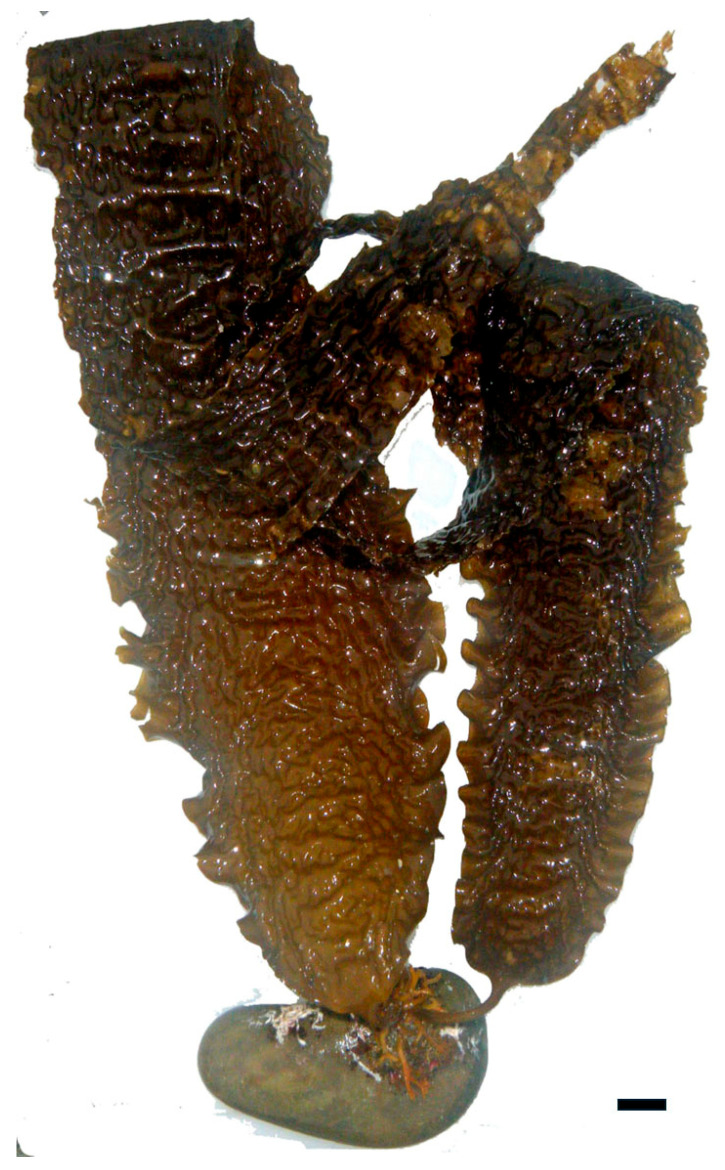
*Saccharina latissima* (Phaeophyceae), a brown alga and key source of laminarin—a storage β-glucan with β(1→3)/(1→6) linkages. Laminarin promotes the growth of beneficial gut microbes such as *Roseburia* and *Faecalibacterium prausnitzii*, enhancing butyrate production and supporting gut epithelial integrity (Scale = 1 cm) (images from the authors).

Laminarin, a storage β-glucan found in *Saccharina latissima* (Figure 18) and *Laminaria digitata*, is another brown algal polysaccharide with notable prebiotic effects. It promotes the growth of SCFA-producing bacteria such as *Roseburia* and *Faecalibacterium prausnitzii*, which are key players in maintaining gut epithelial integrity and modulating immune responses. Laminarin’s β(1→3)/(1→6) linkages are selectively fermented by these microbes, leading to increased butyrate production—a metabolite known to reduce inflammation and support colonocyte health [94].

**Figure 19:**
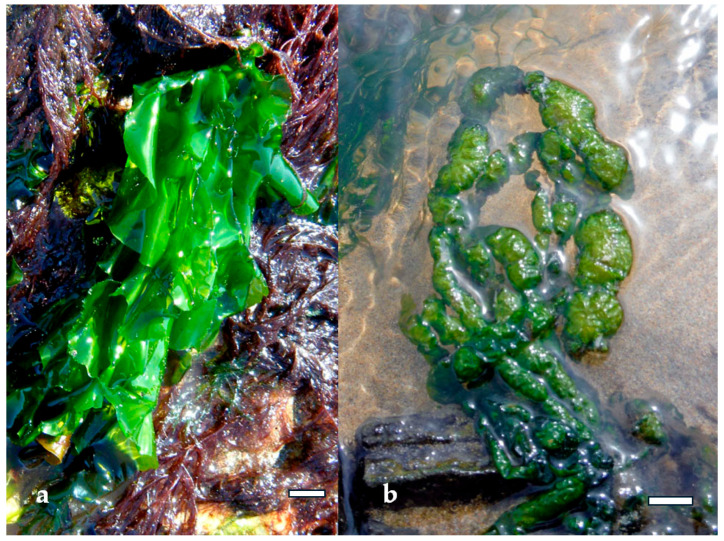
*Ulva rigida* (**a**) and Ulva intestinalis (**b**) (Chlorophyta), green algae species known for producing ulvan—a sulfated polysaccharide structurally similar to mammalian glycosaminoglycans. Ulvan supports gut health by promoting microbial diversity, enhancing populations of *Lactobacillus* and *Bifidobacterium*, and increasing short-chain fatty acid (SCFA) production (Scale = 1 cm) (images from the authors).

Green algae also contribute to gut health through their unique polysaccharides. Ulvan, extracted from *Ulva rigida* (Figure 19a) and *Ulva intestinalis* (Figure 19b), contains rhamnose, xylose, and uronic acids, and its sulfated structure resembles mammalian glycosaminoglycans. Ulvan has been shown to increase microbial diversity and support populations of *Lactobacillus* and *Bifidobacterium*, while also enhancing SCFA production. Its antioxidant and anti-inflammatory properties further contribute to gut homeostasis by reducing oxidative stress and supporting epithelial barrier function [95].

**Figure 20:**
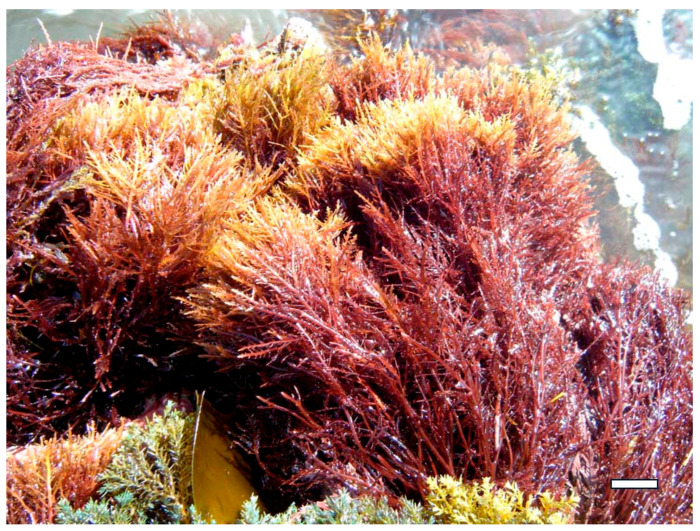
*Gelidium corneum* (Rhodophyta), a red alga used in the extraction of agarose—a slowly fermented polysaccharide linked to increased production of acetate and propionate. These short-chain fatty acids contribute to lipid metabolism and immune regulation, highlighting the prebiotic potential of red algal polysaccharides (Scale = 1 cm) (image from the authors).

Red algae, though less commonly associated with prebiotic research, offers intriguing possibilities. Porphyran, derived from *Neopyropia yezoensis* and *N. tenera*, has demonstrated selective fermentation by Bacteroides species and may contribute to anti-inflammatory effects in the gut. Agarose, from *Gelidium corneum* (Figure 20), is slowly fermented and has been associated with increased levels of acetate and propionate, which play roles in lipid metabolism and immune regulation [96].

Seaweed-derived polysaccharides from a wide range of species—including *A. nodosum*, *F. serratus*, *S. latissima*, *U. rigida*, and *N. yezoensis*—offer diverse and complementary benefits to gut microbiota [4]. Their ability to selectively nourish beneficial microbes, enhance SCFAs production, and support mucosal immunity positions them as valuable components in functional foods, nutraceuticals, and therapeutic strategies aimed at restoring microbial balance and improving host resilience to infection. Continued research into species-specific fermentation profiles, microbial interactions, and clinical outcomes will be essential for unlocking their full potential in gut-targeted health interventions. For instance, note that for many polysaccharides, the precise molecular interactions with viral proteins or bacterial membranes are not fully elucidated and represent an area for future research [97,98].

## 4. Antibacterial Activity of Seaweed Polysaccharides

### 4.1. Summary of In Vitro and In Vivo Studies

The antibacterial potential of seaweed-derived polysaccharides has been increasingly validated through a growing body of in vitro and in vivo studies. These investigations have demonstrated that polysaccharides from red, brown, and green macroalgae demonstrated significant inhibition against a wide range of pathogenic bacteria, including both Gram-positive and Gram-negative strains. Their mechanisms of action include disruption of bacterial membranes, inhibition of biofilm formation, interference with quorum sensing, and modulation of host immune responses (Table 1) [25,89].

**Figure 21:**
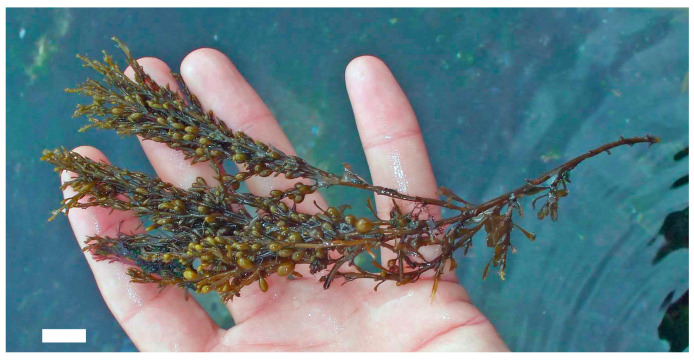
*Sargassum muticum* (Phaeophyceae), a brown alga used in the extraction of fucoidan—a highly sulfated, branched polysaccharide with demonstrated antibacterial activity. Fucoidan from this species disrupts bacterial membranes and biofilms, contributing to its inhibitory effects against *Staphylococcus aureus*, *Escherichia coli*, and *Pseudomonas aeruginosa* (Scale = 1 cm) (image from the authors).

In vitro studies have provided compelling evidence of direct antibacterial activity. For example, fucoidan extracted from *F. vesiculosus* and *Sargassum muticum* (Figure 21) has shown strong demonstrated significant inhibition against *Staphylococcus aureus*, *Escherichia coli*, and *Pseudomonas aeruginosa* [8,40]. These effects are attributed to fucoidan’s high sulfate content and branched structure, which enables it to interact with bacterial cell walls, increase membrane permeability, and ultimately lead to cell lysis [102]. Similarly, ulvan from *U. lactuca* has demonstrated dose-dependent inhibition of *Bacillus subtilis* and *Listeria monocytogenes*, with its sulfated rhamnose residues playing a key role in disrupting bacterial adhesion and biofilm integrity [103].

Carrageenan, particularly the κ- and λ-types derived from *K. alvarezii* and *Sarcopeltis skottsbergii* (formerly *Gigartina skottsbergii*), has been shown to inhibit the growth of *Helicobacter pylori* and *Streptococcus mutans* in vitro. These findings suggest potential applications in gastrointestinal and oral health, where carrageenan may serve as a natural antimicrobial agent in functional foods or dental formulations [104,105]. Agarose from *Gelidium amansii* has also exhibited antibacterial activity, particularly when combined with other bioactive compounds, enhancing its efficacy against *Enterococcus faecalis* and *Salmonella typhimurium* [104].

In vivo studies have further substantiated these findings, demonstrating the therapeutic relevance of seaweed polysaccharides in animal models. Oral administration of fucoidan from *U. pinnatifida* in mice infected with *E. coli* resulted in reduced bacterial load in the intestines and improved survival rates, accompanied by enhanced expression of antimicrobial peptides and reduced inflammatory cytokines [76,105]. Another study involving ulvan supplementation in rats showed a significant decrease in intestinal colonization by *Clostridium perfringens*, along with increased levels of beneficial gut bacteria and improved mucosal immunity [106].

Alginate from *Macrocystis pyrifera* has been evaluated in wound healing models, where its incorporation into hydrogel dressings led to accelerated healing and reduced bacterial contamination. The carboxyl groups in alginate contribute to their ability to chelate essential ions and create an unfavorable environment for bacterial growth, while its gel-forming properties provide a physical barrier against infection [107].

Collectively, these in vitro and in vivo studies highlight the broad-spectrum antibacterial activity of seaweed-derived polysaccharides and underscore their potential as natural alternatives to synthetic antimicrobials. Their biocompatibility, biodegradability, and multifunctional properties make them attractive candidates for integration into pharmaceutical, nutraceutical, and biomedical applications. Continued research into species-specific activity, synergistic effects with other compounds, and clinical validation will be essential for translating these findings into effective therapeutic solutions [108,109].

### 4.2. Potential Applications in Medicine and Food Preservation

The antibacterial properties of seaweed-derived polysaccharides have opened promising avenues for their application in both medicine and food preservation. Their natural origin, biocompatibility, and broad-spectrum activity against pathogenic bacteria make them attractive alternatives to synthetic antimicrobials, particularly in the context of rising antibiotic resistance and consumer demand for clean-label products [110].

In medical applications, seaweed polysaccharides are being explored for use in wound care, drug delivery systems, and infection prevention. Alginate, extracted from brown algae such as *M. pyrifera* and *L. hyperborea*, is widely used in wound dressings due to its gel-forming ability, moisture retention, and capacity to inhibit bacterial colonization [111]. Alginate-based hydrogels can be loaded with antimicrobial agents or used alone to create a physical barrier that prevents infection while promoting tissue regeneration. Fucoidan from *F. vesiculosus* and *S. muticum* has shown potential in topical formulations for treating skin infections and ulcers, owing to its ability to disrupt bacterial membranes and modulate inflammatory responses [112]. Additionally, carrageenan from *K. alvarezii* has been incorporated into nasal sprays and vaginal gels as a barrier against viral and bacterial pathogens, demonstrating efficacy in reducing transmission of respiratory and sexually transmitted infections [113].

In drug delivery, seaweed polysaccharides serve as carriers for antibiotics and bioactive compounds, enhancing their stability, bioavailability, and targeted release. For example, laminarin from *L. digitata* has been used in nanoparticle formulations to deliver antimicrobial peptides directly to infected tissues, improving therapeutic outcomes while minimizing systemic side effects [114]. Ulvan from *U. lactuca* is being investigated for its potential in oral delivery systems, where its sulfated structure can protect encapsulated drugs from enzymatic degradation and facilitate absorption in the gut [115].

In the field of food preservation, seaweed polysaccharides offer natural and effective solutions for extending shelf life and ensuring microbial safety. Their incorporation into edible films and coatings has shown significant promise. For instance, fucoidan and alginate have been used to create antimicrobial packaging materials that inhibit the growth of *Listeria monocytogenes*, *Salmonella enterica*, and *Escherichia coli* on fresh produce, seafood, and meat products. These films not only act as physical barriers but also release antimicrobial agents in response to moisture or temperature changes, providing dynamic protection during storage and transport [116].

Carrageenan and agar from red algae such as *S. skottsbergii* and *Gelidium corneum* have also been employed in dairy and bakery products to prevent spoilage and enhance texture. Their gelling properties allow for uniform dispersion of antimicrobial compounds, while their natural origin aligns with consumer preferences for additive-free and sustainable ingredients [105,117,118]. Moreover, the antioxidant activity of certain polysaccharides, such as porphyran from *Pyropia haitanensis*, contributes to lipid stabilization and color retention in processed foods, further enhancing product quality [119].

## 5. Antiviral Activity of Seaweed Polysaccharides

### 5.1. Overview of Studies Demonstrating Antiviral Properties

The antiviral activity of seaweed-derived polysaccharides has been extensively documented across a wide range of species, highlighting their potential as natural agents for the prevention and treatment of viral infections. These polysaccharides—particularly those with high degrees of sulfation—exhibit broad-spectrum antiviral effects against both enveloped and non-enveloped viruses, including herpesviruses, retroviruses, orthomyxoviruses, papillomaviruses, and coronaviruses (see Table 2). Their mechanisms of action include inhibition of viral attachment and entry, suppression of replication, and modulation of host immune responses [120].

Table 2 provides a comparative summary of the antimicrobial efficacy of sulfated polysaccharides extracted from different seaweed species. Notably, polysaccharides from red algae such as carrageenan and porphyran exhibit strong activity against Gram-positive bacteria, while fucoidan from brown algae demonstrates broader-spectrum effects, including antiviral properties. These findings underscore the structural diversity and therapeutic potential of seaweed-derived polysaccharides, supporting their candidacy as natural antimicrobial agents.

**Figure 22:**
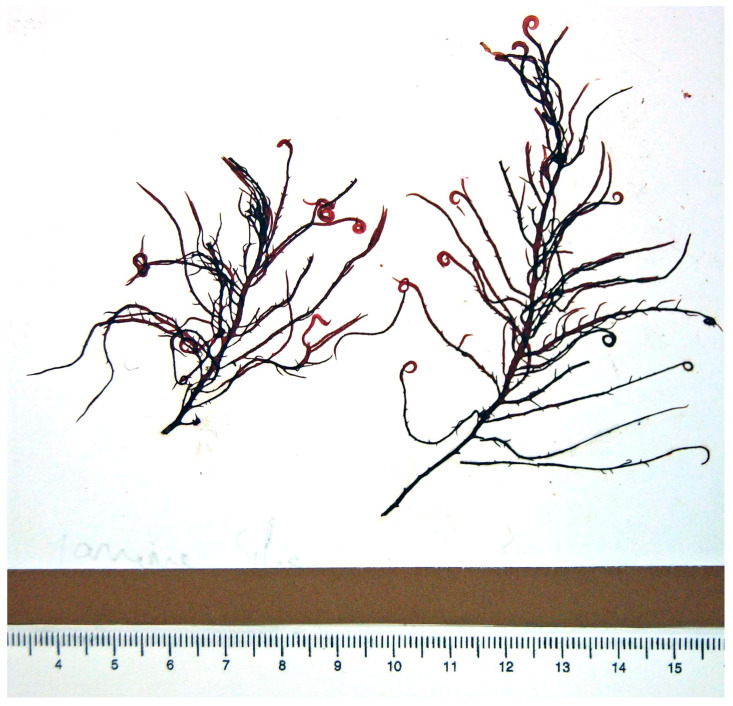
*Hypnea musciformis* (Rhodophyta), a red alga known for its production of lambda- and iota-carrageenan—sulfated polysaccharides with potent antiviral properties. Carrageenan from this species inhibits viral adsorption and internalization, contributing to its efficacy against human rhinovirus, influenza A virus, dengue virus, and HPV (image from the authors).

Among red algae, *S. skottsbergii* and *Hypnea musciformis* (Figure 22) have yielded lambda-carrageenan with potent antiviral effects [144]. In vitro studies have demonstrated that iota-carrageenan inhibits human rhinovirus and influenza A virus by preventing viral adsorption and internalization [145]. Similarly, carrageenan from *H. musciformis* has shown efficacy against dengue virus and HPV, acting as a competitive inhibitor at the cell surface. These findings have led to the development of carrageenan-based nasal sprays and topical gels, which have demonstrated protective effects in clinical trials against respiratory and sexually transmitted viruses [146].

Brown algae continue to be a rich source of antiviral polysaccharides. *Sargassum fusiforme*, for example, produces fucoidan with demonstrated activity against hepatitis B virus (HBV) and enterovirus 71 [43]. In vitro assays revealed that fucoidan from *S. fusiforme* inhibits viral replication and enhances interferon signaling pathways [70]. Another species, *Turbinaria decurrens*, has yielded fucoidan fractions that suppress HIV-1 replication by interfering with reverse transcriptase and integrase enzymes. In vivo studies using mouse models have shown that oral administration of fucoidan from *T. decurrens* reduces viral load and improves survival rates, suggesting systemic immunomodulatory effects [68].

Green algae such as *Ulva rigida* and *Codium fragile* have also demonstrated antiviral potential. Ulvan extracted from *U. rigida* has shown demonstrated significant inhibition against vesicular stomatitis virus and Newcastle disease virus, primarily through its sulfated rhamnose and uronic acid residues that block viral attachment [147]. *C. fragile*, known for its sulfated galactans, has exhibited activity against herpes simplex virus and RSV. These polysaccharides not only interfere with viral entry but also stimulate the production of antiviral cytokines such as interferon alpha (IFN-α) and interleukin 12 (IL-12), enhancing host immune defenses [148].

Other red algae such as *Pyropia haitanensis* and *Gracilariopsis lemaneiformis* have yielded porphyran and agar-type polysaccharides with antiviral properties. Porphyran from *P. haitanensis* has demonstrated activity against enteroviruses and rotavirus, with studies showing reduced viral replication and improved epithelial barrier function [149]. Agar-type polysaccharides from *G. lemaneiformis* have shown potential against influenza virus, especially when combined with zinc or other trace elements that enhance antiviral efficacy [150].

Even less commonly studied species like *Padina pavonica* (brown algae) and *Gymnogongrus flabelliformis* (formerly *Ahnfeltiopsis flabelliformis*) (red algae) have contributed to the growing evidence base [151]. Fucoidan from *P. pavonica* has shown activity against herpes viruses, while sulfated galactans from *Dictyopteris polypodioides* (brown algae) have demonstrated inhibition of HIV-1 entry in vitro [152].

The antiviral properties of seaweed-derived polysaccharides span a diverse array of species and viral targets. From *Gymnogongrus griffithsiae* (Rhodophyta) to *Ulva rigida* (Chlorophyta), these marine biopolymers offer a multifaceted approach to viral inhibition—combining direct interference with viral life cycles and enhancement of host immune responses. Their natural origin, structural diversity, and biocompatibility make them promising candidates for integration into antiviral therapies, prophylactic formulations, and functional foods. Continued research into species-specific activity, molecular mechanisms, and clinical validation will be essential to fully harness their therapeutic potential [153].

### 5.2. Mechanisms Targeting Viral Adsorption, Replication, and Immune Modulation

The antiviral efficacy of seaweed-derived polysaccharides is largely attributed to their ability to interfere with key stages of the viral life cycle—namely, adsorption, replication, and immune modulation. These mechanisms are closely linked to the structural features of the polysaccharides, particularly their degree of sulfation, molecular weight, and monosaccharide composition, which enable them to interact with viral particles and host cell receptors in a highly specific manner [119].

Inhibition of viral adsorption is one of the most well-documented mechanisms. Sulfated polysaccharides such as λ-carrageenan from *S. skottsbergii* and *H. musciformis*, and fucoidan from *F. vesiculosus* and *S. fusiforme*, mimic host cell surface glycosaminoglycans (GAGs), which are commonly exploited by viruses for initial attachment [22]. By competitively binding to viral envelope glycoproteins, these polysaccharides prevent the virus from interacting with its natural receptors on host cells, thereby blocking entry. For example, λ-carrageenan has demonstrated potent inhibition of HPV, HSV, and influenza A virus in vitro, while fucoidan has shown similar effects against HIV-1 and HBV [154].

**Figure 23:**
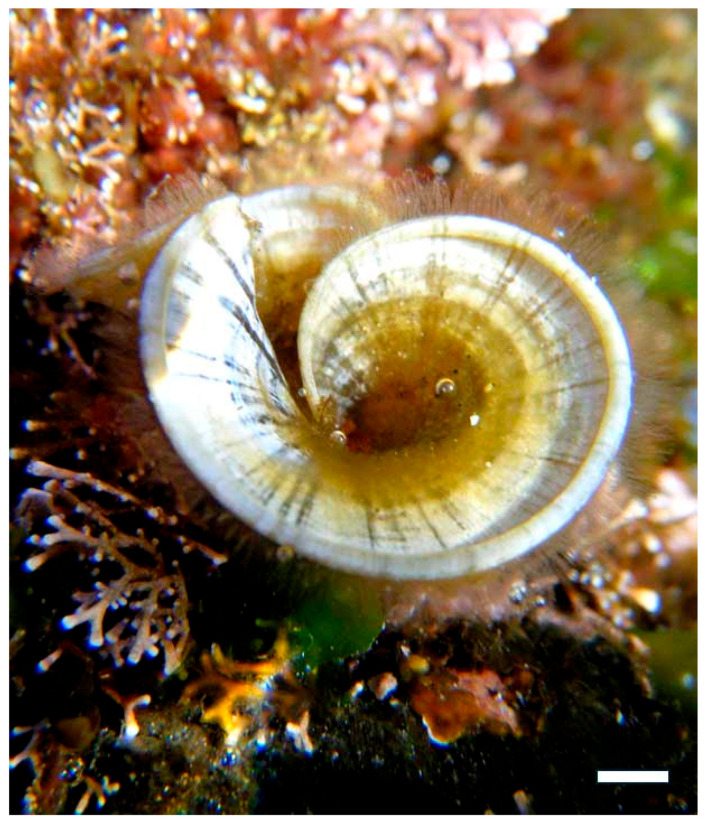
*Padina pavonica* (Phaeophyceae), a brown alga known for its fucoidan content. Fucoidan from this species interferes with retroviral enzymes such as reverse transcriptase and integrase, contributing to the suppression of viral replication (Scale = 1 cm) (image from the authors).

Suppression of viral replication occurs through several pathways. Fucoidan from *Turbinaria ornata* and *Padina pavonica* (Figure 23) has been shown to interfere with reverse transcriptase and integrase enzymes in retroviruses, thereby halting the replication process [22]. Porphyran from *P. haitanensis* has demonstrated the ability to inhibit viral RNA synthesis and reduce the expression of viral proteins in enterovirus-infected cells [155]. In some cases, polysaccharides may also disrupt the assembly and release of viral particles, as observed with ulvan from *Ulva rigida*, which interferes with vesicular stomatitis virus replication by destabilizing the viral envelope and inhibiting budding [95].

Immune modulation is a complementary mechanism that enhances the host’s antiviral defenses. Polysaccharides such as laminarin from *Laminaria digitata* and *Saccharina japonica* activate innate immune receptors like Dectin-1 and TLRs, leading to the production of antiviral cytokines such as IFN-α, IL-12, and TNF-α. These cytokines promote the activation of NK cells, macrophages, and cytotoxic T lymphocytes, which are essential for clearing infected cells and controlling viral spread [156]. Fucoidan has also been shown to enhance antigen presentation by increasing the expression of MHC class I and II molecules, thereby bridging innate and adaptive immunity [157].

Ulvan from *U. intestinalis* further contributes to immune modulation by promoting mucosal immunity. Its structural similarity to mammalian glycosaminoglycans allows it to interact with epithelial cells and stimulate the secretion of secretory IgA and antimicrobial peptides, reinforcing the barrier function of mucosal surfaces. This is particularly relevant in respiratory and gastrointestinal infections, where mucosal immunity plays a critical role in preventing viral colonization [157,158].

Seaweed-derived polysaccharides exert antiviral effects through a multifaceted approach: they block viral adsorption by mimicking host receptors, suppress replication by interfering with viral enzymes and genome synthesis, and enhance immune responses through cytokine induction and antigen presentation. These mechanisms, often act synergistically, underscore the therapeutic potential of marine polysaccharides as natural antivirals. Their broad-spectrum activity, low toxicity, and biocompatibility make them promising candidates for integration into prophylactic and therapeutic formulations aimed at controlling viral infections [47,119].

## 6. Extraction, Purification, and Commercial Applications

### 6.1. Current Methods for Isolation and Processing

The extraction and purification of seaweed-derived polysaccharides are critical steps that influence their yield, structural integrity, and bioactivity. These processes vary depending on the type of polysaccharide, the species of seaweed, and the intended application—whether for pharmaceutical, nutraceutical, cosmetic, or food industry use. Optimizing these methods is essential for ensuring consistency, scalability, and functional performance in commercial formulations [159].

Hot water extraction remains the most widely used technique for isolating polysaccharides such as laminarin, ulvan, and agar. For example, laminarin from *Laminaria digitata* and *Saccharina japonica* is typically extracted by heating dried biomass in distilled water at temperatures ranging from 80 to 100 °C, followed by filtration and ethanol precipitation. This method preserves the β-glucan structure and yields a product suitable for immunomodulatory and antioxidant applications [104]. Similarly, ulvan from *Ulva rigida* and *Ulva intestinalis* is extracted using hot water or dilute acid (e.g., HCl or acetic acid), with subsequent purification steps including centrifugation, dialysis, and freeze-drying to retain its sulfated heteropolysaccharide profile [160].

Alkaline extraction is commonly employed for carrageenan and alginate, which require more robust conditions to release cell wall-bound polysaccharides. Carrageenan from *K. alvarezii* and *Eucheuma denticulatum* is extracted using potassium hydroxide or sodium hydroxide at elevated temperatures, followed by filtration, alcohol precipitation, and drying. The type of carrageenan—kappa, iota, or lambda—is determined by the species and extraction conditions, which influence its gelling properties and antiviral potency [161].

Alginate from *Macrocystis pyrifera* and *A. nodosum* is similarly extracted using alkaline solutions, then converted to sodium alginate through acid precipitation and neutralization. The resulting polymer is widely used in wound dressings, drug delivery systems, and food packaging due to its gel-forming and biocompatible properties [35].

Enzymatic extraction is gaining attention as a more selective and environmentally friendly approach. Enzymes such as cellulases, proteases, and agarases are used to degrade non-polysaccharide components and facilitate the release of target molecules [162]. For instance, enzymatic treatment of *Gracilariopsis lemaneiformis* enhances agar yield and purity, while preserving its gelling capacity. This method reduces the need for harsh chemicals and minimizes structural degradation, making it suitable for high-value biomedical applications [163].

Ultrasound-assisted and microwave-assisted extraction techniques have also been developed to improve efficiency and reduce processing time. These methods use physical energy to disrupt cell walls and enhance solvent penetration, increasing polysaccharide yield and preserving bioactivity [163]. For example, microwave-assisted extraction of fucoidan from *Sargassum ilicifolium* has been shown to improve sulfate retention and antioxidant capacity, while reducing thermal degradation [164,165].

Purification typically involves a combination of precipitation (using ethanol or acetone), dialysis, ultrafiltration, and chromatographic techniques. Ethanol precipitation is widely used to concentrate polysaccharides and remove low-molecular-weight impurities. Dialysis and ultrafiltration help eliminate salts and small molecules, while chromatographic methods—such as ion-exchange, gel filtration, and affinity chromatography—enable fractionation based on charge, size, or specific binding properties. These techniques are critical for obtaining high-purity polysaccharides suitable for biomedical and industrial application [165].

Dialysis and ultrafiltration help eliminate salts and small molecules, while ion-exchange chromatography can be employed to separate polysaccharide fractions based on charge density—particularly useful for sulfated compounds like fucoidan and carrageenan. A study demonstrated that fucoidan extracted from *Gongolaria barbata* (Phaeophyceae) exhibits potent anticandidal activity against *Candida albicans*, *C. glabrata*, and *C. parapsilosis*. Notably, a minimum fungicidal concentration of just 0.1 μg/mL was sufficient to inhibit the growth of all tested *Candida* species [166].

In commercial settings, these extraction and purification methods are scaled up using industrial reactors, membrane filtration systems, and spray-drying technologies to produce standardized polysaccharide powders or gels. Quality control measures—including molecular weight analysis, sulfate content determination, and microbial testing—are essential to ensure product consistency and safety [167].

Notably, the structure-activity paradox, where subtle variations in molecular architecture can lead to markedly different biological outcomes, poses both a challenge and an opportunity in therapeutic design. Understanding these nuanced relationships is essential for tailoring polysaccharide-based interventions to target specific microbial threats with precision and efficacy.

The isolation and processing of seaweed polysaccharides involve a diverse array of techniques tailored to the structural characteristics of each compound and the functional requirements of its application. Advances in green extraction technologies, coupled with precision purification strategies, are paving the way for sustainable and high-quality production of marine biopolymers for use in medicine, food preservation, and beyond [168]. Green extraction methods, such as enzymatic hydrolysis, ultrasound-assisted extraction (UAE), and microwave-assisted extraction (MAE), are considered environmentally friendly because they typically operate at lower temperatures, require less solvent, and significantly reduce energy consumption and chemical waste compared to conventional solvent-based approaches. These techniques not only improve extraction efficiency but also align with the principles of green chemistry, making them ideal for scalable and eco-conscious biopolymer production [168].

### 6.2. Industrial-Scale Applications and Market Potential

The industrial-scale application of seaweed-derived polysaccharides has expanded significantly in recent years, driven by growing demand for sustainable, bioactive ingredients across multiple sectors—including pharmaceuticals, nutraceuticals, cosmetics, agriculture, and food packaging. Their multifunctional properties, biocompatibility, and renewable origin position these marine biopolymers as strategic assets in the global bioeconomy, with market projections indicating robust growth in the coming decade [169].

In the pharmaceutical industry, seaweed polysaccharides such as fucoidan, carrageenan, alginate, and ulvan are being developed as active ingredients in antiviral, antibacterial, anti-inflammatory, and immunomodulatory formulations [3]. Fucoidan from *F. vesiculosus* and *S. fusiforme* is being incorporated into oral supplements and injectable therapies for immune support and viral inhibition, particularly in the context of respiratory and chronic viral infections [170]. Carrageenan from *S. skottsbergii* is already commercialized in nasal sprays and vaginal gels for its barrier-forming and antiviral properties, with clinical trials supporting its efficacy against influenza and HPV [143]. Alginate from *Macrocystis pyrifera* is widely used in wound dressings, drug delivery systems, and tissue engineering scaffolds, owing to its gel-forming ability and biocompatibility [74]. Ulvan from *U. lactuca* is gaining traction in regenerative medicine and vaccine adjuvant development due to its structural similarity to mammalian glycosaminoglycans [59].

In the nutraceutical and functional food sectors, seaweed polysaccharides are marketed for their prebiotic, antioxidant, and metabolic health benefits. Laminarin and fucoidan are included in dietary supplements targeting gut health, immune modulation, and metabolic regulation [171,172]. Porphyran from *P. haitanensis* is used in antioxidant-rich formulations, while ulvan is being explored for its potential to support microbiota diversity and intestinal barrier function [173]. The global market for seaweed-based nutraceuticals is projected to grow substantially, driven by consumer interest in natural, plant-based health solutions and the increasing prevalence of lifestyle-related diseases [174].

The cosmetic industry has embraced seaweed polysaccharides for their moisturizing, anti-aging, and photoprotective properties [175]. Carrageenan and alginate are used as stabilizers and texture enhancers in creams and serums, while fucoidan and ulvan are valued for their bioactivity in skin repair and inflammation control [176]. Products containing *A. nodosum* extracts are marketed for their anti-wrinkle and skin-brightening effects, and formulations with *U. rigida* polysaccharides are promoted for their antioxidant and soothing properties [177].

In agriculture, seaweed polysaccharides are used in biostimulants and soil conditioners to enhance plant growth, stress tolerance, and microbial activity [178]. Alginate and laminarin-based formulations improve water retention and nutrient uptake [179], while ulvan has shown promise in inducing plant defense responses against pathogens [180]. These applications align with the shift toward sustainable and organic farming practices, offering eco-friendly alternatives to synthetic agrochemicals [181].

**Figure 24:**
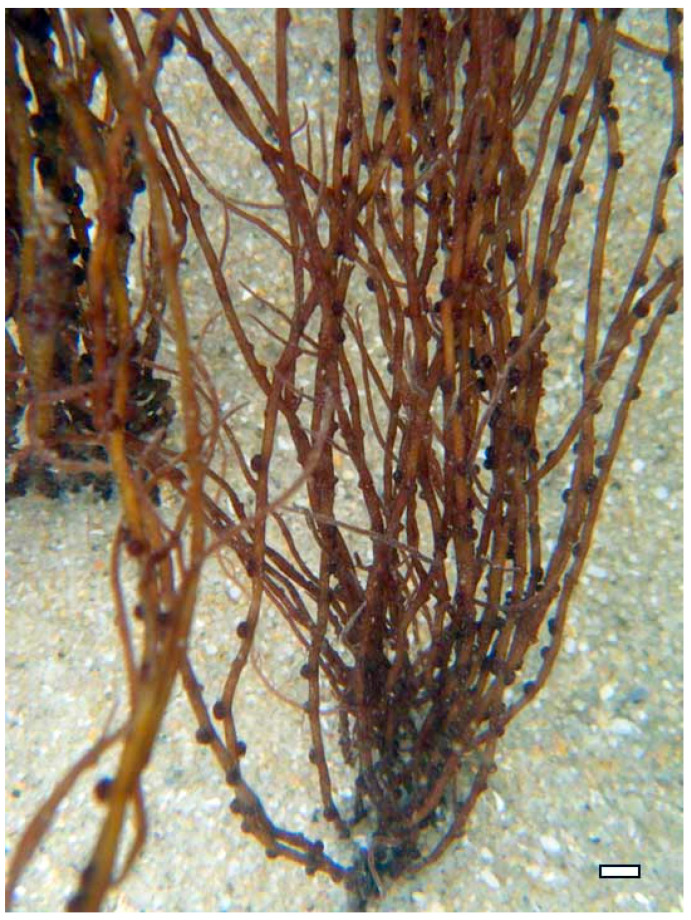
*Gracilaria gracilis* (Rhodophyta), a red alga used in the development of biodegradable food packaging. Agar extracted from this species is incorporated into edible coatings that enhance microbial inhibition and preserve sensory quality in fresh produce, seafood, and meat products (Scale = 1 cm) (image from the authors).

In food packaging and preservation, polysaccharides such as alginate, carrageenan, and fucoidan are used to develop biodegradable films and coatings with antimicrobial properties. These materials extend shelf life, reduce spoilage, and meet regulatory and consumer demands for plastic-free packaging [182]. Edible coatings incorporating *Gracilaria gracilis* (Rhodophyta) (Figure 24) agar or *Sargassum angustifolium* fucoidan are being tested for use on fresh produce, seafood, and meat products, with promising results in microbial inhibition and sensory quality retention [183,184].

The potential market for seaweed-derived polysaccharides is substantial. According to industry reports, the global seaweed extract market is expected to surpass USD 4.8 billion by 2035, with polysaccharides representing a significant share of this growth. Factors driving this expansion include increased investment in marine biotechnology, favorable regulatory frameworks for natural ingredients, and rising consumer awareness of sustainability and health. Europe, Asia-Pacific, and North America are leading regions in commercial development, with Portugal, France, South Korea, and Japan emerging as innovation hubs for seaweed cultivation and processing [174].

The industrial-scale applications of seaweed polysaccharides span a diverse array of sectors, each leveraging their unique structural and functional properties. Their market potential is reinforced by global trends in health, sustainability, and bio-based innovation, positioning these marine biopolymers as key contributors to the future of green technology and natural therapeutics [185].

## 7. Challenges and Future Perspectives

This review offers a comprehensive and integrative perspective on the therapeutic potential of seaweed-derived polysaccharides, with a specific focus on their antimicrobial and antiviral properties. Unlike previous reviews that primarily catalog the structural features or industrial applications of these compounds, our work emphasizes their biomedical relevance in the context of rising antimicrobial resistance and emerging viral threats. We uniquely synthesize recent findings on both in vitro and in vivo bioactivities, elucidate mechanisms of action, including membrane disruption and viral entry inhibition, and highlight advances in extraction and purification that enhance therapeutic efficacy. Furthermore, we present a comparative overview (Table 1) of polysaccharide-specific antimicrobial profiles, which has not been systematically addressed in earlier literature. By bridging molecular insights with translational applications, this review positions seaweed polysaccharides as promising candidates for next-generation functional foods and biopharmaceuticals.

## 8. Conclusions

The exploration of seaweed-derived polysaccharides has revealed their exceptional potential as multifunctional bioactive compounds with wide-ranging applications in human health and sustainable innovation. This review has emphasized the structural diversity of polysaccharides extracted from red, brown, and green macroalgae—such as carrageenan, fucoidan, laminarin, alginate, ulvan, agarose, and porphyran—and demonstrated how their chemical composition, sulfation patterns, and molecular architecture underpin a broad spectrum of biological activities [186]. These compounds exhibit potent antibacterial and antiviral properties, acting through mechanisms that include inhibition of pathogen adhesion, disruption of microbial membranes, suppression of viral replication, and modulation of host immune responses. Their ability to mimic host–cell surface receptors and activate both innate and adaptive immunity positions them as promising candidates for therapeutic applications targeting infectious diseases [187,188].

In addition to their antimicrobial effects, seaweed polysaccharides contribute significantly to gut health through their prebiotic activity. By selectively stimulating beneficial microbial populations and enhancing short-chain fatty acid production, compounds such as laminarin, fucoidan, ulvan, and porphyran support intestinal barrier integrity, reduce inflammation, and promote systemic immune resilience [188]. Advances in extraction and purification techniques, including hot water, alkaline, enzymatic, and microwave-assisted methods, have enabled scalable production while preserving bioactivity, facilitating their integration into pharmaceuticals, nutraceuticals, cosmetics, agriculture, and food packaging [189].

Industrial applications continue to expand, with seaweed polysaccharides being incorporated into wound dressings, drug delivery systems, functional foods, biodegradable films, and plant biostimulants [169]. Their biocompatibility, renewable sourcing, and multifunctional properties align with global trends in sustainability and natural product innovation. The market potential for these compounds is substantial, supported by increasing consumer demand, favorable regulatory frameworks, and ongoing investment in marine biotechnology [190].

Looking forward, future research should focus on elucidating structure–activity relationships at the molecular level, conducting clinical trials to validate safety and efficacy, developing synergistic formulations with conventional therapeutics, and establishing standardized protocols for quality control and regulatory compliance [191]. Seaweed-derived polysaccharides represent a powerful convergence of marine science, biotechnology, and health innovation. Their continued development and application hold promise not only for improving human health but also for advancing sustainable solutions across multiple sectors [17].

Despite the growing body of evidence supporting the therapeutic potential of seaweed-derived polysaccharides, several challenges remain that must be addressed to fully harness their biomedical and commercial value. One of the primary limitations is the structural complexity and variability of these compounds, which can differ significantly depending on species, geographic origin, seasonal variation, and extraction method. This heterogeneity complicates reproducibility in experimental studies and hinders the development of standardized formulations [192]. Future research must prioritize the establishment of robust analytical frameworks to characterize molecular weight, sulfation patterns, and monosaccharide composition with precision, enabling clearer correlations between structure and bioactivity [193].

Another challenge lies in the scalability and sustainability of extraction and purification processes. While conventional methods such as hot water and alkaline extraction are widely used, they often involve high energy consumption, chemical waste, and potential degradation of bioactive components. There is a pressing need to develop green and cost-effective technologies, such as enzyme-assisted, ultrasound, or microwave-based extraction, which preserve functional integrity while minimizing environmental impact. Integrating biorefinery approaches that valorize the entire seaweed biomass, including pigments, proteins, and minerals, could enhance economic viability and reduce waste [194].

From a biomedical perspective, the lack of clinical validation remains a significant barrier. Most studies to date have been conducted in vitro or in animal models, and while these provide valuable insights, they do not fully capture the complexity of human physiology. Rigorous clinical trials are essential to confirm safety, efficacy, dosage parameters, and long-term effects of seaweed polysaccharides in humans [17]. Additionally, regulatory harmonization across regions is needed to facilitate market entry and consumer confidence, especially for applications in pharmaceuticals and functional foods [195,196].

There are also opportunities for innovation in formulation science. The development of targeted delivery systems, such as nanoparticles, hydrogels, and encapsulated matrices, could enhance the bioavailability and therapeutic precision of these compounds. Combining seaweed polysaccharides with other bioactive compounds, including probiotics, polyphenols, or conventional drugs, may yield synergistic effects that improve outcomes in infection control, immune modulation, and gut health [197,198].

Moreover, the exploration of underutilized and endemic seaweed species offers a vast frontier for discovery. Many marine ecosystems, particularly in the Atlantic and Indo-Pacific regions, harbor unique macroalgae with uncharacterized polysaccharide profiles. Bioprospecting efforts, coupled with genomic and metabolomic tools, could uncover novel compounds with superior bioactivity and industrial relevance [27].

In conclusion, while challenges persist in standardization, scalability, and clinical translation, the opportunities for future research are equally compelling. By advancing interdisciplinary collaborations across marine biology, biotechnology, pharmacology, and materials science, the full potential of seaweed-derived polysaccharides can be realized—contributing to innovative, sustainable solutions for global health and industry [199,200,201].

## Figures and Tables

**Table 1:** Antibacterial activity of Sulfated polysaccharides in some bacterial infections.

Sulfated Polysaccharide	Source	Dose/IC_50_ (µg mL^−1^)	Bacterial Infection and Effect of Treatment	Ref.
Fucoidan	*Fucus vesiculosus* (brown algae)	IC_50_: 250 µg/mL	Inhibits *Staphylococcus aureus* and *Escherichia coli*; disrupts biofilm formation	[99]
Methanolic extract	*Ecklonia cava* (brown algae)	IC_50_: 256 μg/mL	Effective against *Listeria monocytogenes*; reduces bacterial adhesion	[100]
Carrageenan (κ-type)	*Kappaphycus alvarezii* (red algae)	IC_50_: 150 µg/mL	Suppresses *Pseudomonas aeruginosa* growth and biofilm formation	[101]
Ulvan	*Ulva lactuca* (green algae)	IC_50_: 300 µg/mL	Inhibits *Salmonella typhimurium*; interferes with quorum sensing	[101]
Sulfated galactan	*Gracilariopsis longissima* (red algae)	IC_50_: 180 µg/mL	Active against *Bacillus subtilis* and *E. coli*; damages cell membrane integrity	[99]
Alginic acid	*Macrocystis pyrifera* (brown algae)	IC_50_: 220 µg/mL	Inhibits *Vibrio cholerae* and *E. coli*; interferes with cell wall synthesis	[100]
Sulfated rhamnan	*Monostroma nitidum* (green algae)	IC_50_: 160 µg/mL	Effective against *Streptococcus pyogenes*; inhibits bacterial proliferation	[101]
Porphyran	*Neopyropia yezoensis* (red algae)	IC_50_: 190 µg/mL	Inhibits *Helicobacter pylori*; disrupts membrane potential	[99]
Sulfated xylogalactan	*Eucheuma denticulatum* (red algae)	IC_50_: 210 µg/mL	Active against *Klebsiella pneumoniae*; reduces biofilm formation	[100]
Sulfated arabinogalactan	*Codium fragile* (green algae)	IC_50_: 170 µg/mL	Targets *Enterococcus faecalis*; inhibits cell division	[101]
Fucoidan	*Fucus vesiculosus* (brown algae)	IC_50_: 250 µg/mL	Inhibits *Staphylococcus aureus* and *Escherichia coli*; disrupts biofilm formation	[99]
Laminarin sulfate	*Laminaria digitata* (brown algae)	IC_50_: 200 µg/mL	Effective against *Listeria monocytogenes*; reduces bacterial adhesion	[100]
Carrageenan (κ-type)	*Kappaphycus alvarezii* (red algae)	IC_50_: 150 µg/mL	Suppresses *Pseudomonas aeruginosa* growth and biofilm formation	[101]
Ulvan	*Ulva lactuca* (green algae)	IC_50_: 300 µg/mL	Inhibits *Salmonella typhimurium*; interferes with quorum sensing	[101]
Sulfated galactan	*Gracilaria verrucosa* (red algae)	IC_50_: 180 µg/mL	Active against *Bacillus subtilis* and *E. coli*; damages cell membrane integrity	[99]
Alginic acid	*Macrocystis pyrifera* (brown algae)	IC_50_: 220 µg/mL	Inhibits *Vibrio cholerae* and *E. coli*; interferes with cell wall synthesis	[100]
Sulfated rhamnan	*Monostroma nitidum* (green algae)	IC_50_: 160 µg/mL	Effective against *Streptococcus pyogenes*; inhibits bacterial proliferation	[101]
Porphyran	*Porphyra yezoensis* (red algae)	IC_50_: 190 µg/mL	Inhibits *Helicobacter pylori*; disrupts membrane potential	[99]
Sulfated xylogalactan	*Eucheuma denticulatum* (red algae)	IC_50_: 210 µg/mL	Active against *Klebsiella pneumoniae*; reduces biofilm formation	[100]
Sulfated arabinogalactan	*Codium fragile* (green algae)	IC_50_: 170 µg/mL	Targets *Enterococcus faecalis*; inhibits cell division	[101]
Fucoidan	*Fucus vesiculosus* (brown algae)	IC_50_: 250 µg/mL	Inhibits *Staphylococcus aureus* and *Escherichia coli*; disrupts biofilm formation	[99]

**Table 2:** Antiviral activity of carrageenan and fucoidan in some virus infections.

Sulfated Polysaccharide	Source	Dose/IC_50_ (µg mL^−1^)	Virus Infection and Effect of Treatment	Refs.
Fucoidan	*Undaria pinnatifida* (P)	In vivo study with 5 mg day^−1^ twice a day for 14 days	Anti-IAV activity; Positive effect on production of antigen-specific antibody; Inhibition of virus attachment and blocking virus penetration	[121,122,123]
	*Kjellmaniella crassifolia* (P)	250 µg mL^−1^ of fucoidan with purity of 92.8%	Inhibition of influenza A virus infection; targeting viral neuraminidase	[124]
	*Saccharina japonica* (P)	50–500 µg mL^−1^ of fucoidan with 1.9% of uronic acids and 10.4% of sulfur in sulfate semi-esters	Antiviral activity against avian influenza A (H5N1) virus infection in the cultured cells	[125]
	*Saccharina* *cichorioides*, *S.* *japonica* (P)	0.001–100 µg mL^−1^	Anti-HIV activity. Prevention of attachment and cell-to-cell virus spread	[126]
	*Sargassum mcclurei*, *Sargassum polycystum*, *Turbinaria ornata* (P)	IC_50_ value 0.33–0.7 µg mL^−1^	Fucoidans blocking the early steps of HIV entry into target cells	[127]
	*Sargassum swartzii* (P)	1.56 and 6.25 μg mL^−1^	Fucoidan fractions exhibit significant anti-HIV-1 activity	[128]
	*Cladosiphon okamuranus* (P)	0.83 g day^−1^	Anti-HCV activity. Inhibits virus replication	[129]
	*Scytosiphon lomentaria* (P)	IC_50_ value 0.76- 1.34 µg mL^−1^	Anti-HSV activity; The galactofucan fractions of fucoidan showed antiviral activity because of the low uronic acid and high sulfate esters content; Inhibition of virus attachment	[130]
	*Sargassum henslowianum* (P)	IC_50_ value 0.89 and 0.82 µg mL^−1^		[131]
	*Fucus distichus* subsp. *evanescens* (P)	In vitro study with 0.25–250 µg mL^−1^ In vivo study with 10 mg kg^−1^ day^−1^	Antivirus activity against HSV, ECHO-1, and HIV-1; Inhibiting virus replication	[132]
Carrageenan	Commercial carrageenan	Iota-carrageenan	Anti-IAV; Inhibition virus replication	[133]
	κ-carrageenan	Kappa carrageenan and sulfated derivatives; in vivo study with 40 mg kg^−1^ d^−1^	Inhibition virus replication	[134]
	κ-carrageenan	Kappa, acetylated and sulfated derivatives; in vivo study with 30 mg kg^−1^ day^−1^	Carrageenan displayed higher activity than Rabivirin at the dose of 30 mg/kg·d	[135]
	Lambda-carrageenan	Lambda-carrageenan IC_50_ 1–20 ng mL^−1^	Anti-HPV potential; Inhibition of virus attachment and blocking virus penetration	[136]
	Iota-carrageenan	Carrageenan and Zanamivir act in vitro synergistically against several influenza A virus strains (H1N1(09)pdm, H3N2, H5N1, H7N7).	Nasal spray containing only iota-carrageenan, or together with “zanamivir” provide treatment of upper respiratory tract infections in patients under suspicion of infection by influenza A (H1N1)	[82,137]
	*Sarcopeltis* *Skottsbergii* (R)	Lambda-carrageenan IC_50_ 0.52 and 10.42 for BoHv-1 and SuHV-1, respectively	BoHV-1 and SuHV-1; Inhibition of virus attachment and blocking virus penetration	[138]
	*Stenogramma* *Interruptum* (R)	Kappa/iota and lambda-carrageenans 0.65–2.88 µg mL^−1^	Anti-HSV activity; Inhibition of virus attachment and blocking virus penetration	[139]
	*Sarcopeltis* *Skottsbergii* (R)	Lambda-carrageenan 10 mg mL^−1^	interfere with protein binding to the heparan sulfate coreceptor in host tissues	[140]
	*Sarcothalia* *Atropurpurea* (R)	Kappa and lambda-carrageenan 0.2–0.8 µg mL^−1^	Interfere with protein binding to the heparan sulfate co-receptor in host tissues	[141]
	*Solieria chordalis* (R)	Anti-*Herpes simplex* virus (HSV-1) activity	Anti-*Herpes simplex* virus (HSV-1) activity	[142]
	*Solieria filiformis* (R)	Iota-carrageenan 4.5–11.7 µg mL^−1^	Anti-*Herpes simplex* virus (HSV-1) activity	[143]

P—Phaeophyceae; R—Rhodophyta.

## Data Availability

No new data were created or analyzed in this study. Data sharing is not applicable to this article.

## Abbreviations

The following abbreviations are used in this manuscript:

BoHV-1	Bovine alphaherpesvirus 1
ECHO-1	Enteric cytopathic human orphan virus 1
HBV	Hepatitis B virus
HCV	Hepatitis C virus
HIV	Human immunodeficiency viruses
HPV	Human papillomavirus
HSV	Herpes simplex virus
H5N1	Influenza A virus subtype (Avian influenza)
IAV	Influenza A virus
IL-6	Interleukin 6
IL-12	Interleukin 12
IFN-α	Interferon alpha
IFN-γ	Interferon gamma
MAE	Microwave-assisted extraction
MHC	Major histocompatibility complex
NK	Natural killer cells
PRRs	Pattern recognition receptors
RNA	Ribonucleic acid
RSV	Respiratory syncytial virus
SARs	Structure–activity relationships
SCFAs	Short-chain fatty acids
SuHV	Suid herpes virus 1
TLRs	Toll-like receptors
TNF-α	Tumor necrosis factor-alpha
UAE	ultrasound-assisted extraction
